# Embryonic stem cell-derived extracellular vesicles delay cellular senescence by inhibiting oxidative stress

**DOI:** 10.1016/j.jbc.2025.110821

**Published:** 2025-10-14

**Authors:** Shun Enomoto, Yun Ha Hur, Tatiana Solodova, Jacob Neumann, Richard A. Cerione, Marc A. Antonyak

**Affiliations:** 1 Department of Molecular Medicine, Cornell University, Ithaca, New York, USA; 2 Department of Life Sciences, Pohang University of Science and Technology, Pohang, Republic of Korea; 3 Department of Chemistry and Chemical Biology, Cornell University, Ithaca, New York, USA

**Keywords:** cell signaling, cellular senescence, extracellular vesicles, exosome, fibronectin, glycogen synthase kinase 3 (GSK-3), microvesicles, oxidative stress, reactive oxygen species (ROS)

## Abstract

The accumulation of cells that permanently exit the cell cycle and undergo senescence is a hallmark of aging and predisposes organisms to disease. Emerging evidence suggests extracellular vesicles (EVs) released by pluripotent embryonic stem cells (ESCs) possess therapeutic/regenerative properties with the potential to significantly impact cellular senescence and aging-related disorders. However, the critical next step for taking advantage of the potential benefits offered by ESC-derived EVs will be to unravel the molecular mechanisms responsible for their unique functional effects, which thus far have not been fully defined. Toward that goal, we now identify a signaling pathway essential for EVs shed by ESCs to potently block fibroblasts and astrocytes from undergoing senescence. It starts with the extracellular matrix protein fibronectin that coats the surfaces of EVs, enabling the vesicles to bind integrins on cells and trigger the activation of FAK and AKT. This leads to the inhibition of GSK3β activity and stabilization of the transcription factor Nrf2, which counteracts the effects of oxidative stress that would otherwise drive cellular senescence. These findings define a signaling pathway used by ESC-derived EVs to extend cellular lifespan, highlighting their potential application in anti-aging strategies.

Aging is characterized by the gradual decline in physiological processes, resulting in organisms with impaired tissue function and a propensity to develop deadly diseases, including cancer and neurodegeneration (1, 2, 3). One of the most consistent characteristics of aging is the induction of cellular senescence, a process in which cells undergo irreversible cell cycle arrest (4, 5, 6). Many cellular stresses, including the continuous passaging (growth) of cells, the build-up of reactive oxygen species (ROS), and oncogene activation, have been reported to induce senescence (7, 8, 9). Metabolic stress can drive cells to undergo senescence and contribute to the production of secretomes that contain unique sets of factors collectively referred to as the senescence-associated secretory phenotype (SASP) (10). SASP can promote the formation of fibrotic and inflammatory local environments that contribute to the onset and progression of pathologies, including several types of cancer, Alzheimer’s disease, and cardiovascular disorders (11). Thus, approaches aimed at delaying senescence or counteracting the effects of the SASP are considered to be potential strategies for extending lifespans as well as preventing aging-associated diseases (12, 13, 14).

Embryonic stem cells (ESCs) are derived from the inner cell masses of blastocyst-stage embryos, and they possess two properties that distinguish them from virtually all other cell types (15, 16). One is their ability to differentiate into every cell lineage in the body, while the other is their capability for continuously generating more pluripotent stem cells, a process referred to as self-renewal (17). Indeed, ESCs are one of the few cell types that are typically far less prone to undergo senescence. In addition to their essential roles in early development, these unique traits of ESCs, together with those of other types of stem cells, have attracted significant attention from basic and translational researchers, as well as the pharmaceutical industry, due to their potential use in a wide range of regenerative medicine applications (18, 19, 20). Specifically, stem cells are being heavily investigated as sources to replace dysfunctional adult cells within aged, diseased, and damaged tissues.

Small membrane-enclosed particles that are formed and released by stem cells, generally referred to as extracellular vesicles (EVs), are also being examined for their regenerative capabilities (21, 22, 23, 24). EVs are most often classified into two major subgroups, microvesicles (MVs) and exosomes (25, 26). MVs, which are also known as ectosomes, range from 200 to 800 nm in diameter and are produced by the outward budding and fission of the plasma membrane, while exosomes, or small EVs (sEVs), are 30 to 150 nm in size and arise from intraluminal vesicles within multi-vesicular bodies (MVBs). In the endocytic pathway, MVBs are typically trafficked to lysosomes, where their contents are degraded (27). However, some MVBs are directed to the cell surface, where they fuse with the plasma membrane and release their contents, including exosomes, into the extracellular space (28). Both MVs and exosomes contain several types of cargo, including proteins and nucleic acids within their lumen or bound to their outer surfaces (25, 26). EVs and their associated cargo can be transferred to recipient cells and induce important phenotypic changes (29, 30, 31, 32, 33, 34, 35). For example, we recently showed that MVs and exosomes produced by ESCs play a critical role in maintaining their pluripotency, as well as promoting the implantation of the embryo into the uterus (36, 37). Other groups have reported using stem cell-derived EVs for regenerative medicine applications, where these vesicles were shown to promote the recovery of damaged skin and heart tissue in animal models, and to reduce the extent of liver and kidney fibrosis (38, 39). Recent work has also shown that EVs isolated from plasma taken from young individuals were able to rejuvenate aged tissues by inhibiting the accumulation of senescent cells and suppressing mitochondrial dysfunction (40).

Despite the promising therapeutic and regenerative potential of stem cell-derived EVs, the underlying signaling mechanisms responsible for their exciting functional capabilities are still poorly defined. Here, we demonstrate that EVs produced by pluripotent ESCs effectively block various cell types, including fibroblasts and astrocytes, from undergoing senescence. We show that ESC-derived EVs give rise to these striking outcomes due to the extracellular matrix protein, fibronectin, which is bound to their outer surfaces, activating a specific integrin-dependent signaling pathway within the cells. These signals halt the build-up of ROS, enabling the EV-treated cells to continue to grow under conditions where they would have otherwise exited the cell cycle and undergone senescence.

## Results

### ESCs produce large amounts of MVs and exosomes that can be transferred to fibroblasts

To establish that EVs produced by pluripotent ESCs prevent differentiated adult cells from undergoing senescence, E14tg2α.4 ESCs and primary mouse embryonic fibroblasts (MEFs) were used (Fig. S1*A*). The ESCs expressed several different pluripotent markers, including the master regulators of pluripotency Oct3/4 and Nanog, as well as SOX2 (41) (Fig. S1*B*, lane labeled *ESCs*). They were also capable of robustly forming spheres (42) and exhibited high levels of alkaline phosphatase (AP) activity (43), as expected for pluripotent stem cells (Fig. S1, *C*–*F*, images and bars labeled *ESCs*); whereas, MEFs lacked expression of the pluripotent markers but expressed the membrane glycoprotein and fibroblast marker Thy1 (44) (Fig. S1*B*, lane labeled *MEFs*). Moreover, MEFs failed to form spheres and showed little AP activity (Fig. S1, *C*–*F*, images and bars labeled *MEFs*).

We compared the ability of ESCs and MEFs to undergo senescence using replicative exhaustion, which involves passaging the cells until they no longer are able to divide (4, 5). Each cell type was serially passaged for a few weeks before being examined for senescence-associated β-galactosidase (SA-β-gal) activity (45) using a pH-dependent colorimetric assay that results in senescent cells turning blue. After a total of eight passages (P8), approximately 50% of the MEFs stained positive for SA-β-gal activity (Fig. S1, *G* and *H*, images and bars labeled *MEFs*), whereas very few ESCs showed SA-β-gal activity after undergoing the same number of passages (P8) or even following as many as 25 passages (P25) (Fig. S1, *G* and *H*, images and bars labeled *ESCs*). These findings highlight how ESCs undergo self-renewal, whereas MEFs are sensitive to replicative exhaustion and undergo senescence.

**Figure 1:**
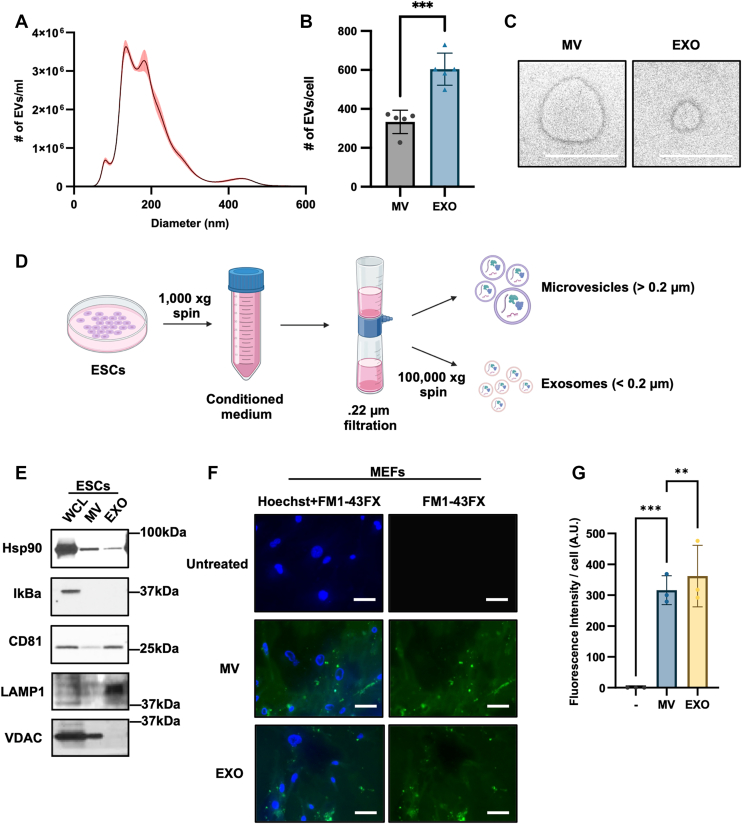
**ESCs produce large amounts of MVs and exosomes that can be transferred to fibroblasts.***A*, the number and sizes of EVs present in the conditioned medium collected from pluripotent ESCs (5 × 10^3^ cells/cm^2^) was determined using Nanoparticle Tracking Analysis (NTA). The experiment was performed five independent times (n = 5). *B*, the number of MVs (EVs larger than 220 nm) and exosomes (EVs smaller than 220 nm) produced per ESC was determined using the data from (*A*). *C*, negative stain electron microscopy images of MVs and exosomes in ESC conditioned medium. Scale bar = 200 nm. Data is representative of three independent experiments (n = 3). *D*, diagram of the EV isolation procedure. Details of the procedure can be found in Experimental Procedures. *E*, Western blot of MVs and exosomes isolated from ESCs, as well as the ESCs themselves (whole cell lysate; WCL), probed for the MV markers Hsp90 and VDAC, the whole cell marker IΚBα, and the exosome markers CD81 and LAMP1. Data are representative of three independent experiments (n = 3). *F*, images of MEFs treated with MVs and exosomes (EXO) from ESCs labeled with the fluorescent membrane dye FM 1 to 43X for 1 h. The cells were also stained with Hoechst. Scale bar = 20 μm. Data are representative of three independent experiments (n = 3). *G*, fluorescence signal intensity detected in (*F*) was determined using ImageJ software. The data shown in (*B*) and (*G*) were determined using Student’s *t* test; ∗∗∗*p* < 0.001; and ∗∗*p* < 0.01. Shaded areas and error bars indicate means ± standard deviations (SD).

In accordance with the MISEV 2023 recommendations and guidelines regarding EV isolation and characterization (46), we then determined the number and sizes of EVs shed from ESCs by performing nanoparticle tracking analysis (NTA) (36) on their conditioned medium. Figure 1*A* shows that the medium contained secreted particles ranging in diameter between 70 to 500 nm, consistent with the reported sizes for exosomes and MVs (37) (Fig. 1*A*). Based on the size distribution of these particles, we estimate that ESCs produce ∼2-fold more exosomes than MVs (Fig. 1*B*). Negative stain electron microscopy carried out on the conditioned medium showed exosomes and MVs of expected sizes and morphology (47) (Fig. 1*C*).

The MVs and exosomes were isolated from the ESC-conditioned medium using a combination of size filtration and low and high-speed centrifugation (48) (Fig. 1*D*). The MV and exosome preparations, together with the ESCs (whole cell lysates; WCL), were lysed and analyzed by Western blotting. The MV markers, Hsp90 (49) and VDAC (50), were predominantly detected in the MV fraction, while the exosome-specific markers, CD81 (51) and LAMP1 (46), were enriched in the exosome lysate (Fig. 1*E*). The samples were also probed for IκBα (*i.e.*, NF-κB inhibitor α), which was detected only in the cell extracts (WCL), as a control to demonstrate that the MV and exosome preparations do not simply contain every cellular component.

We next determined that EVs produced by pluripotent ESCs were able to associate with fibroblasts. The conditioned medium collected from ESCs was incubated with FM1-43X, a dye capable of fluorescence emission upon intercalating into the lipid membranes of EVs (52). The medium was then subjected to the EV isolation procedure (see Fig. 1*D*) to generate preparations of MVs and exosomes that were fluorescently labeled, while removing any unincorporated free dye. Fibroblasts were either left untreated or were treated with the labeled MVs and exosomes for 1 h, at which point the cells were washed extensively with phosphate-buffered saline (PBS), fixed, stained with Hoechst to detect their nuclei, and visualized using fluorescent microscopy. Fluorescent signals were readily detected associated with cells treated with MVs and exosomes, often appearing as small puncta (Fig. 1, *F* and *G*). On the other hand, no fluorescent signal was detected in the untreated cells.

### EVs derived from ESCs delay cellular senescence

**Figure 2:**
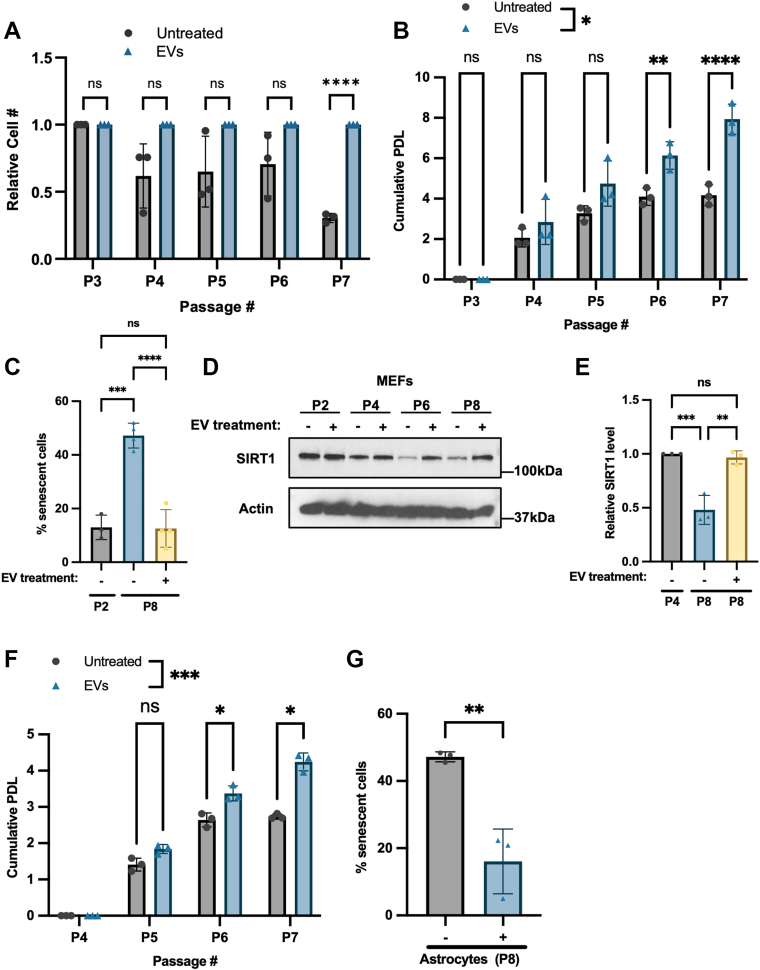
**The EVs derived from ESCs delay cellular senescence.***A*, Passage 3 (P3) MEFs were treated without (untreated) or with MVs and exosomes (EVs) from ESCs. The cells were trypsinized, counted, and re-plated (5 × 10^3^ cells/cm^2^) in medium supplemented without or with EVs every 3 days until they were serially passaged 7 times (P7). The number of cells at each passage was determined. The experiment was performed three independent times (n = 3). *B*, the cumulative population doubling level (PDL) was determined for each condition in (*A*). *C*, β-galactosidase activity assays were performed on MEFs treated without (−) or with (+) EVs isolated from ESCs for two or eight passages (P2 or P8). The experiment was performed four independent times (n = 4). *D*, Western blot of MEFs that had been treated without (−) or with (+) EVs from ESCs at each passage. The cells were probed for the senescence marker SIRT1. Actin was the loading control. Data are representative of three independent experiments (n = 3). *E*, relative SIRT1 expression levels in the indicated lanes in (*D*) were determined using ImageJ software. *F*, Passage 3 (P3) astrocytes were treated without (untreated) or with MVs and exosomes (EVs) from ESCs. The cells were trypsinized, counted, and re-plated (5 × 10^3^ cells/cm^2^) in fresh medium supplemented without or with EVs every 3 days until the MEFs were passaged 7 times (P7). The cumulative PDL was determined for each condition. The experiment was performed three independent times (n = 3). *G*, β-galactosidase activity assays were performed on astrocytes treated without (−) or with (+) EVs isolated from ESCs for eight passages (P8). The experiment was performed three independent times (n = 3). The data shown in (*A*), (*C*), (*E*), and (*G*) were determined using Student’s *t* test; (*B*) and (*F*) were determined using two-way ANOVA with Sidak’s multiple comparison tests; ∗∗∗∗*p* < 0.0001; ∗∗∗*p* < 0.001; ∗∗*p* < 0.01; ∗*p* < 0.05; and ns (not significant). Error bars indicate means ± standard deviations (SD).

We then examined if EVs produced by pluripotent ESCs, when transferred to fibroblasts, affected their ability to undergo replicative exhaustion-induced cellular senescence. Untreated MEFs (*i.e.* control MEFs), or MEFs treated with MVs and exosomes collected from ESCs every 3 days, were serially passaged until the growth of the untreated MEFs was dramatically slowed, which occurred after seven passages (P7) (Fig. 2*A*). The cumulative population doubling level ((PDL); *i.e.*, the number of times the cell population doubled over the course of serial passing of the cells) was also determined and showed that the control MEFs stopped growing between passages six and seven (Fig. 2*B*). Moreover, after seven passages, the untreated cells had enlarged and flattened cell bodies (Fig. S2*A*, image labeled *-*). However, MEFs treated with MVs and exosomes from ESCs continued to grow after seven passages (Fig. 2, *A* and *B*) and their cell size was smaller with a more classic fibroblast-like morphology (53) (Fig. S2*A*, image labeled *+*).

MEFs serially passaged and treated without or with the EVs derived from ESCs were evaluated in two additional sets of experiments. First, senescence-associated β-galactosidase (SA-β-gal) activity was examined. While only a small percentage (∼10%) of actively growing, passage 2 (P2) fibroblasts had detectable SA-β-gal activity (cells stained blue), MEFs that had undergone eight passages showed a marked increase in the percentage (∼50%) of cells with SA-β-gal activity (Fig. S2, *B* and *C*). However, MEFs cultured for the same length of time (8 passages) and treated with EVs exhibited a striking reduction in the number of cells with SA-β-gal activity, similar in appearance to passage two fibroblasts (Fig. S2, *B* and *C*). Cells cultured under these same conditions were also examined for the expression of Sirtuin 1 (SIRT1), an NAD^+^-dependent deacetylase that plays a critical role in regulating senescence and aging (54). SIRT1 expression is downregulated during aging and considered to be a hallmark of senescent cells (55, 56), while its ectopic expression in cells and animal models has been reported to promote longevity (57). Indeed, SIRT1 expression was found to decrease in MEFs from passage 2 (P2) to passage 8 (P8) (Fig. 2, *D* and *E*), whereas SIRT1 levels in cells treated with the EVs shed by ESCs were maintained through several passages. Similar changes in the expression of NAMPT were noted in the MEFs (Fig. S2*C*). Like SIRT1, NAMPT expression has been reported to decrease with age and in cells that have undergone senescence (58, 59, 60).

We next determined whether EVs produced by ESCs could similarly delay the induction of senescence in another adult/differentiated cell type, specifically astrocytes derived from C57B/L6 mice. Cultures of astrocytes were treated without or with EVs isolated from ESCs over multiple passages, until the growth of the untreated cells slowed after seven passages (Fig. 2*F*). However, EV-treated astrocytes at this matched time point continued to grow well (Fig. 2*F*). SA-β-gal assays and Western blot analysis were then performed on these cells to determine whether they underwent senescence. Astrocytes which underwent eight passages and were treated with EVs exhibited far fewer senescent cells, compared to the untreated control group (Fig. S2, *D* and *G*).

A critical question that arises from the above findings is whether treating adult/differentiated cells with EVs from ESCs not only delays senescence but also causes the cells to de-differentiate. Thus, Western blot analysis using lineage-specific markers was performed on MEFs and astrocytes treated with EVs from pluripotent ESCs for seven passages. We found that the expression level of the fibroblast marker Thy1 (44) in MEFs was unchanged by the addition of EVs (Fig. S2*E*). Likewise, the expression of glial fibrillary acidic protein (GFAP), a cytoskeletal protein highly expressed in astrocytes (61), was unchanged in cells treated with EVs (Fig. S2*F*). Collectively, these findings highlight the ability of EVs produced by pluripotent ESCs to delay senescence in differentiated adult cell types without causing the cells to undergo de-differentiation.

### EVs derived from ESCs delay senescence in fibroblasts by activating AKT

**Figure 3:**
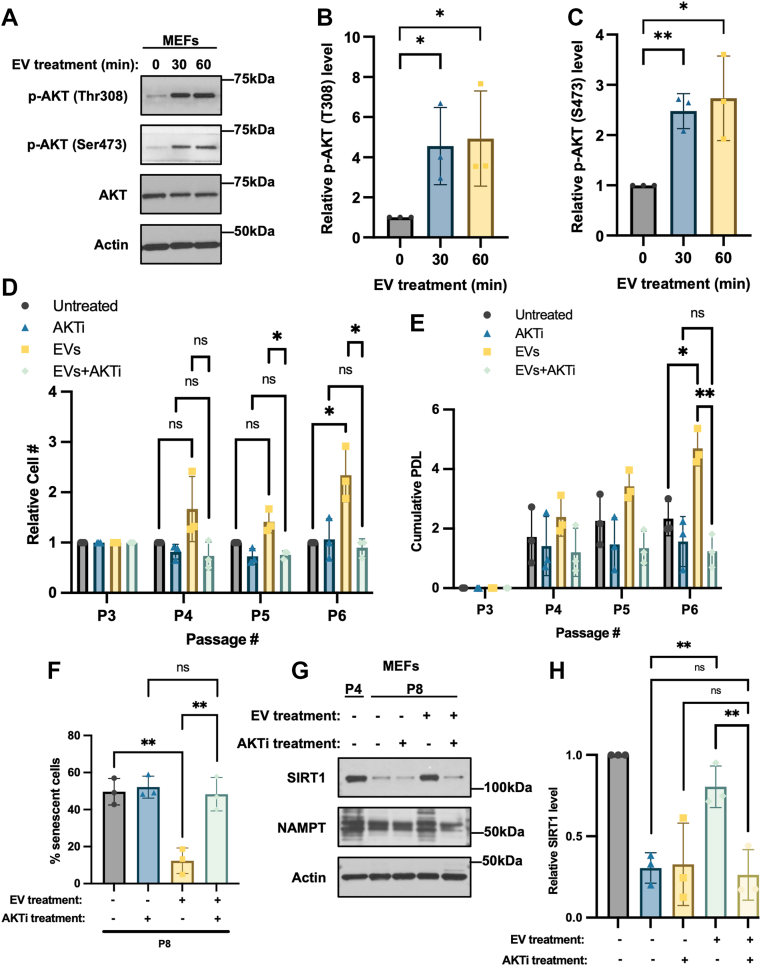
**EVs derived from ESCs delay senescence by activating AKT.***A*, Western blot of serum starved MEFs treated without (−) or with (+) EVs from ESCs for the indicated lengths of time, probed for AKT when it is phosphorylated at Thr 308 (p-AKT (T308)) or Ser 473 (p-AKT (S473)), and total AKT (AKT). Actin was the loading control. Data are representative of three independent experiments (n = 3). *B and C*, relative p-AKT (T308) and p-AKT (S473) levels in (*A*) were determined using ImageJ software. *D*, passage 3 (P3) MEFs were treated without or with MVs and exosomes from ESCs and 1 μM MK-2206 (AKTi). The cells were trypsinized, counted, and re-plated (5 × 10^3^ cells/cm^2^) in fresh medium supplemented without or with EVs and MK-2206 every 3 days until the MEFs were serially passaged 7 times (P7). The number of cells at each passage was determined. The experiment was performed three independent times (n = 3). *E*, the cumulative PDL was determined for each condition in (*D*). *F*, β-galactosidase activity assays were performed on MEFs that had been treated without or with EVs isolated from ESCs and MK-2206 (AKTi) for eight passages (P8). The experiment was performed three independent times (n = 3). *G*, Western blot of Passage 4 (P4) MEFs and MEFs treated without or with EVs from ESCs and MK-2206 (AKTi) for eight passages (P8), probed for the senescence markers SIRT1 and NAMPT. Actin was the loading control. Data are representative of three independent experiments (n = 3). *H*, relative SIRT1 levels in (*G*) were determined using ImageJ software. The data shown in (*B*), (*C*), (*D*), (*F*), and (*H*) were determined using Student’s *t* test; (*E*) was determined using two-way ANOVA with Tukey’s multiple comparison tests; ∗∗*p* < 0.01; ∗*p* < 0.05; and ns (not significant). Error bars indicate means ± standard deviations (SD).

To gain insights into the molecular mechanism and signaling cues used by EVs from pluripotent ESCs to inhibit cellular senescence, we examined whether treating MEFs with ESC-derived EVs affected the activation of key intracellular signaling proteins. One interesting possibility that we considered was AKT, as this serine/threonine kinase is known to play important roles in promoting cell survival, growth, and metabolism (62, 63, 64, 65, 66). Western blot analysis carried out on MEFs treated with both MVs and exosomes isolated from ESCs for increasing lengths of time up to 1 h showed that the EVs triggered a robust increase in the phosphorylation of AKT at two key sites, threonine 308 (Thr308) and serine 473 (Ser473), required for its full activation (62) (Fig. 3, *A*–*C*). We also showed that MEFs treated with either MVs or exosomes isolated from ESCs promoted the phosphorylation of AKT at both Thr308 and Ser473 to a similar extent (Fig. S3, *A* and *B*), suggesting that both classes of EVs were capable of activating AKT.

To establish whether AKT activity was necessary for the ability of EVs to delay senescence, cell growth and SA-β-gal assays were performed on MEFs serially passaged and treated with different combinations of EVs from ESCs together with the AKT inhibitor MK-2206. The ability of the EVs to maintain the growth of serially passaged MEFs and delay senescence (Figs. 3, *D*–*F* and S3*C*) was completely prevented by inhibiting AKT activity. Cells cultured under these conditions were further examined to determine the expression levels of SIRT1 and NAMPT. Figure 3, *G* and *H* show that the ability of MEFs treated with EVs to maintain SIRT1 and NAMPT expression as they were passaged was lost when AKT activity was inhibited.

### Fibronectin coats the surfaces of EVs from ESCs and is important for activating FAK and AKT in recipient cells

**Figure 4:**
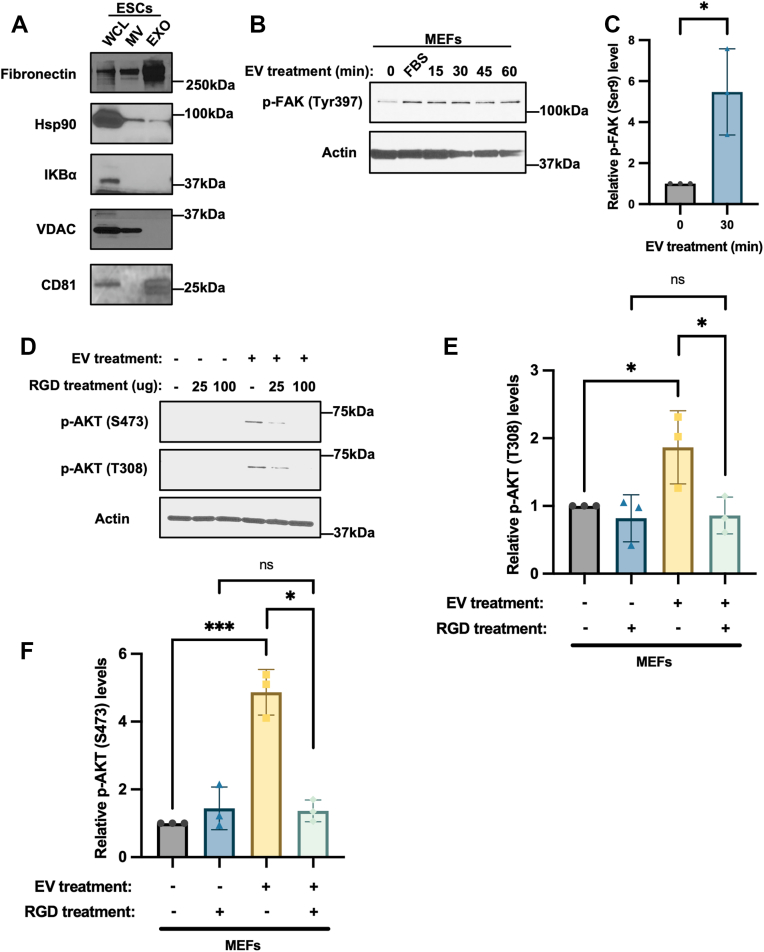
**Fibronectin coats the surfaces of EVs from ESCs and is important for activating FAK and AKT in recipient cells.***A*, Western blot analysis of the MVs and exosomes isolated from ESCs, as well as the ESCs (WCL) themselves, probed for fibronectin, the MV markers Hsp90 and VDAC, the whole cell lysate marker IΚBα, and the exosome marker CD81. Data are representative of three independent experiments (n = 3). *B*, Western blot of serum starved MEFs treated without or with EVs isolated from ESCs for the indicated lengths of time, probed for FAK when it is phosphorylated at Tyr 397 (p-FAK (Y397)). As a positive control, the MEFs were treated with 10% FBS for 1 h. Actin was the loading control. The data are representative of three independent experiments (n = 3). *C*, relative p-FAK (Y397) levels in (*B*) were determined using ImageJ software. *D*, Western blot of serum starved MEFs treated without or with EVs from ESCs and either 25 μg or 100 μg of RGD peptide, probed for AKT when it is phosphorylated at Thr 308 (p-AKT (T308)) or Ser 473 (p-AKT (S473)). Actin was the loading control. The data are representative of three independent experiments (n = 3). *E and F*, Relative p-AKT (T308) and p-AKT (S473) levels for the cells treated with 100 μg of RGD peptide in (*D*) were determined using ImageJ software. The data shown in (*C*), (*E*), and (*F*) were determined using Student’s *t* test; ∗∗∗*p* < 0.001; ∗*p* < 0.05; and ns (not significant). Error bars indicate means ± standard deviations (SD).

We then set out to identify the cargo associated with ESC-derived EVs responsible for stimulating AKT phosphorylation in recipient cells. Fibronectin was considered a primary candidate, since we previously showed that this major extracellular matrix (ECM) protein is associated with MVs and exosomes produced by ESCs (37) (also see Fig. 4*A*) and present along the outer surfaces of the EVs as it was removed when the vesicles were treated with trypsin which digests proteins expressed on their surfaces but not those such as ubiquitinated proteins present within their protective lumen (Fig. S4*A*).

Because fibronectin binds integrins on the plasma membranes of cells and triggers the activation of the non-receptor tyrosine kinase, focal adhesion kinase (FAK), together with its effectors phosphoinositide 3-kinase (PI3K) and AKT (67), we examined whether EVs from ESCs engaged and activated integrins in recipient MEFs. Cells treated with EVs for increasing lengths of time up to 1 h were analyzed to determine the levels of phosphorylated FAK (p-FAK (Tyr397)), as a readout of integrin activation (67, 68). MEFs treated with EVs showed an increase in FAK phosphorylation, compared to untreated cells (Fig. 4, *B* and *C*). To determine whether fibronectin associated with EVs is required for promoting integrin-stimulated AKT phosphorylation, we treated EVs with trypsin to remove fibronectin from their outer surfaces and then examined their ability to promote AKT phosphorylation in MEFs. We found that EVs lacking fibronectin were no longer able to fully promote the phosphorylation of AKT these cells (Fig. S4*B*). We then used the RGD peptide which acts by competing with fibronectin for binding to integrins (69) and found that it blocked the ability of EVs to promote AKT phosphorylation (Fig. 4, *D*–*F*).

### EV-induced FAK activation delays senescence in fibroblasts

**Figure 5:**
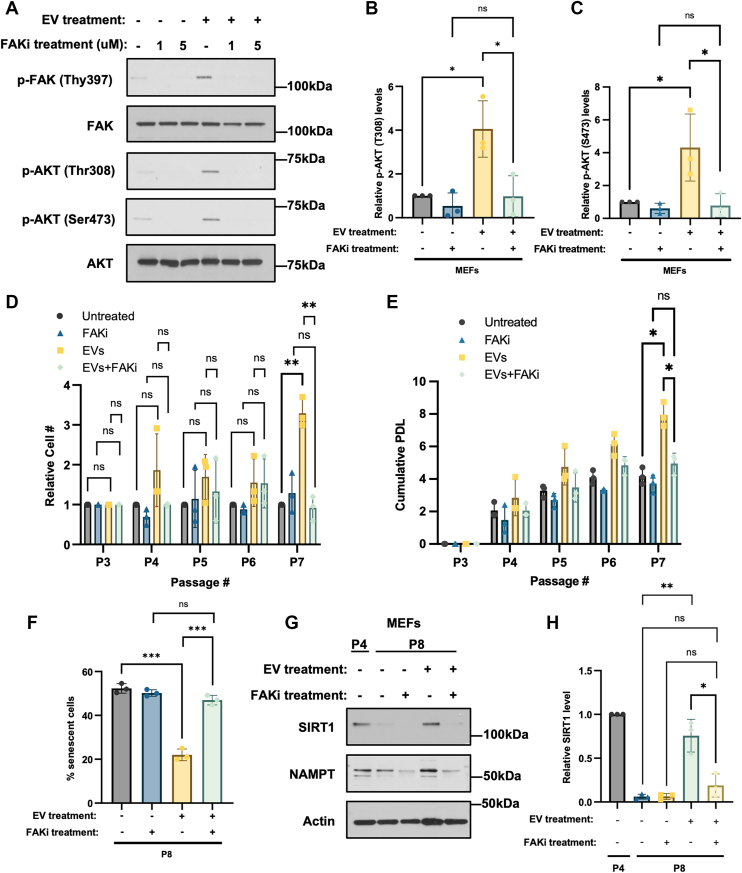
**EV-induced FAK activation delays senescence in fibroblasts.***A*, Western blot analysis of MEFs treated without or with EVs from ESCs and either 1uM or 5 μM FAK inhibitor III (FAKi) for 1 h, probed for phosphorylated FAK (p-FAK (Tyr397)), total FAK (FAK), phosphorylated AKT (p-AKT (T308)), phosphorylated AKT (p-AKT (S473)), and total AKT (AKT). Data are representative of three independent experiments (n = 3). *B and C*, Relative p-AKT (T308) and p-AKT (S473) levels for the cells treated with 1uM FAKi in (*A*) were determined using ImageJ software. *D*, Passage 3 MEFs were treated without or with EVs from ESCs and 5 μM FAK inhibitor III (FAKi). The cells were trypsinized, counted, and re-plated (5 × 10^3^ cells/cm^2^) in fresh medium with EVs and FAK inhibitor III every 3 days until the MEFs were serially passaged 7 times (P7). The number of cells at each passage was determined. The experiment was performed three independent times (n = 3). *E*, cumulative PDL was determined for each condition in (*D*). *F*, β-galactosidase activity assays were performed on MEFs that had been treated without or with EVs from ESCs and FAK inhibitor III for eight passages (P8). The experiment was performed three independent times (n = 3). *G*, Western blot of Passage 4 (P4) MEFs and MEFs treated without or with 5 μM FAK inhibitor III and EVs isolated from ESCs for eight passages (P8), probed for the senescence markers SIRT1 and NAMPT. Actin was the loading control. The data are representative of three independent experiments (n = 3). *H*, Relative SIRT1 expression levels in (*G*) were determined using ImageJ software. The data shown in (*B*), (*C*), (*D*), (*F*), and (*H*) were determined using Student’s *t* test; (*E*) was determined using two-way ANOVA with Tukey’s multiple comparison tests; ∗∗∗*p* < 0.001; ∗∗*p* < 0.01; ∗*p* < 0.05; and ns (not significant). Error bars indicate means ± standard deviations (SD).

Since the phosphorylation of FAK is increased by EV treatment, we asked whether its activity is responsible for increasing AKT activity and delaying senescence. To test this idea, we first assessed the levels of phosphorylated AKT in MEFs treated with EVs from ESCs and a potent FAK inhibitor, FAK inhibitor III (FAKi). Treatment of MEFs with EVs shed by ESCs increased their levels of AKT phosphorylation at Thr308 and Ser473 (Fig. 5, *A*–*C*). However, when MEFs were treated with both EVs and the FAK inhibitor, AKT phosphorylation was reduced to the same levels detected in untreated cells.

MEFs were then treated with different combinations of ESC-derived EVs in the presence and absence of FAK inhibitor III. Figure 5, *D* and *E* show that while serially passaged MEFs treated with EVs continued to grow under conditions where the growth of their untreated counterparts slowed, the addition of the FAK inhibitor completely blocked this growth advantage. SA-β-gal activity assays performed on these same cells also showed that inhibiting FAK activity prevented the EVs from delaying senescence, as the number of senescent cells detected in MEFs treated with EVs and FAK inhibitor III was comparable to what was seen in serially passaged untreated MEFs (Figs. 5*F* and S5). Consistent with these findings, the ability of the EVs to maintain the levels of SIRT1 and NAMPT in serially passaged MEFs was lost when the MEFs were also treated with the FAK inhibitor III (Fig. 5, *G* and *H*).

### EV-stimulated AKT activity inhibits GSK3β activity and the induction of senescence

**Figure 6:**
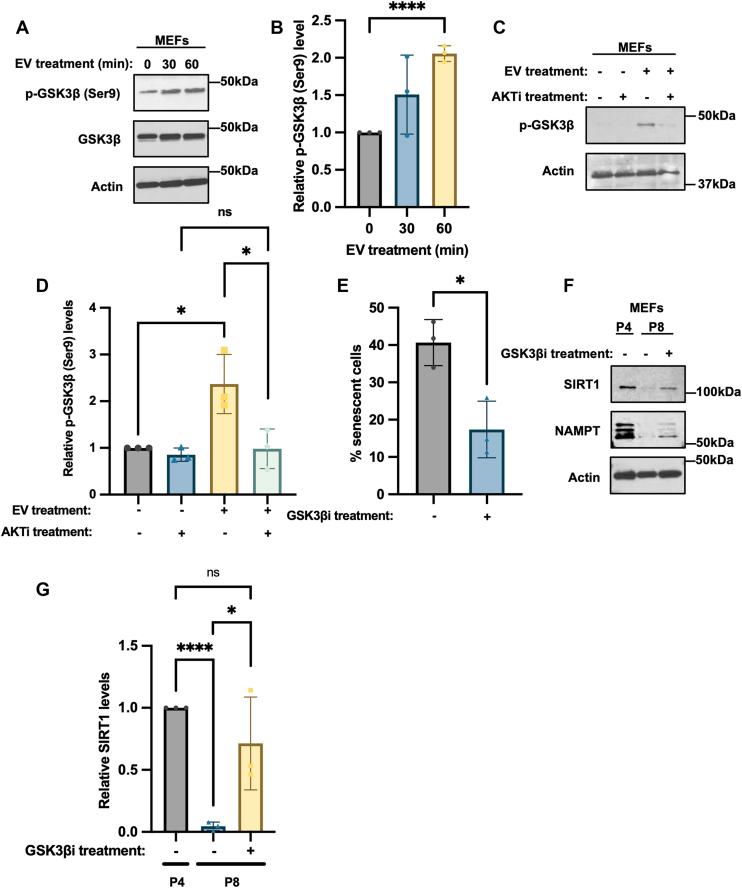
**EV-stimulated AKT activity inhibits GSK3β activity and the induction of senescence.***A*, Western blot of serum starved MEFs treated without or with EVs from ESCs for the indicated lengths of time, probed for GSK3β when it is phosphorylated at Ser nine (p-GSK3β (Ser9)) and total GSK3β (GSK3β). Actin was the loading control. The data are representative of three independent experiments (n = 3). *B*, relative p-GSK3β (Ser9) levels in (*A*) were determined using ImageJ software. *C*, Western blot of serum starved MEFs treated without or with EVs from ESCs and 1uM MK-2206 (AKTi), probed for p-GSK3β (Ser9) and GSK3β. Actin was the loading control. The data are representative of three independent experiments (n = 3). *D*, relative p-GSK3β (Ser9) levels in (*C*) were determined using ImageJ software. *E*, β-galactosidase activity assays were performed on MEFs treated without or with 3 μM GSK3β inhibitor CHIR99021 (GSK3βi) for eight passages (P8). The experiment was performed three independent times (n = 3). *F*, Western blot of MEFs treated without or with 3 μM CHIR99021 for four and eight passages (P4 and P8), probed for the senescence markers SIRT1 and NAMPT. Actin was the loading control. The data are representative of three independent experiments (n = 3). *G*, relative SIRT1 expression levels in (*F*) were determined using ImageJ software. The data shown in (*B*), (*D*), (*E*), and (*G*) were determined using Student’s *t* test; ∗∗∗∗*p* < 0.0001; ∗*p* < 0.05; and ns (not significant). Error bars indicate means ± standard deviations (SD).

Among the known substrates of AKT that might be responsible for mediating its ability to delay cellular senescence, glycogen synthase kinase three beta (GSK3β) appeared to be an attractive candidate. GSK3β has been shown to be involved in regulating a number of important cellular processes, including cell cycle progression (66, 70, 71), and to induce senescence by promoting the degradation of several transcription factors necessary for sustaining cell growth (66). AKT phosphorylates GSK3β at serine 9 (Ser9) and inhibits its kinase activity (70). Thus, we examined whether EVs isolated from ESCs affected the phosphorylation and kinase activity of GSK3β. MEFs treated with EVs for up to 1 h were shown to increase GSK3β phosphorylation without affecting its expression (Fig. 6, *A* and *B*). The increase in GSK3β phosphorylation by EVs was abolished when treating the cells with the AKT inhibitor MK-2206 (Fig. 6, *C* and *D*). Likewise, the ability of EVs to enhance the phosphorylation of GSK3β was also blocked by treatment with the RGD peptide, as well as with FAK inhibitor III (Fig. S6, *A* and *B*), further demonstrating the importance of the integrin-FAK-AKT pathway in promoting the phosphorylation of GSK3β by EVs.

We then examined whether the inhibition of GSK3β activity was sufficient to delay senescence. MEFs treated without or with CHIR99021, a potent GSK3β inhibitor, were serially passaged. Treatment with the GSK3β inhibitor delayed senescence as read-out by β-gal activity assays and maintained the expression levels of SIRT1 and NAMPT (Figs. 6, *E*–*G* and S6*C*).

### EVs from ESCs upregulate the expression of Nrf2 to inhibit ROS levels in recipient cells

**Figure 7:**
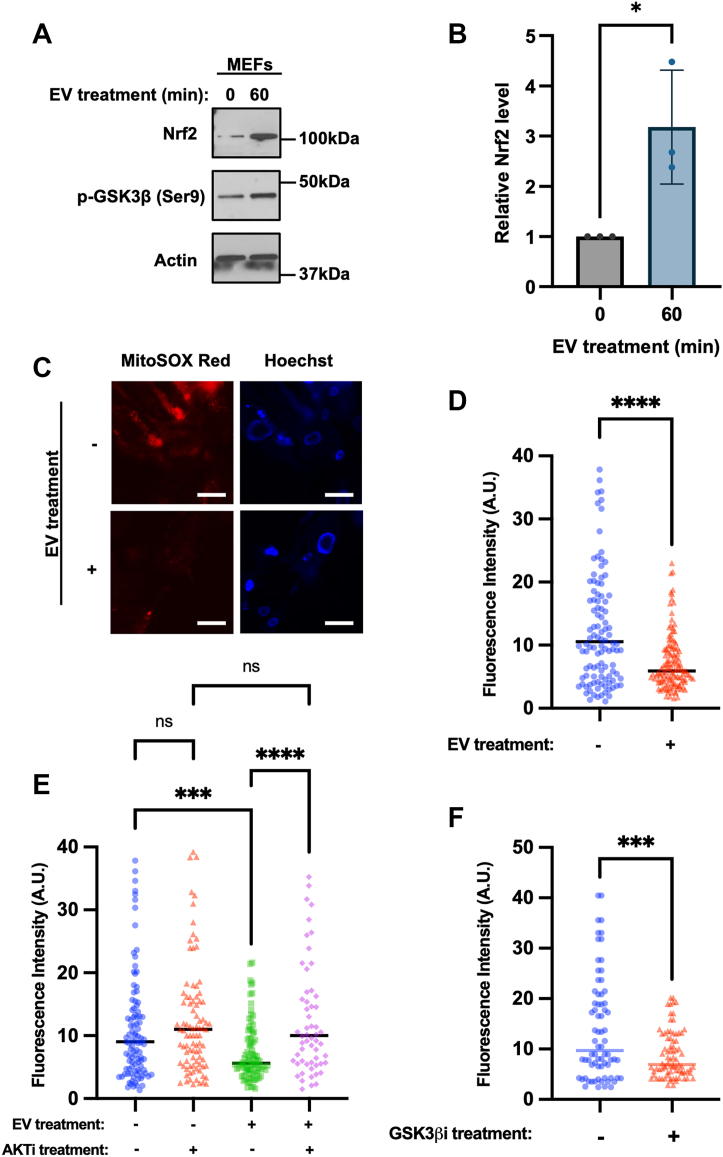
**EVs from ESCs upregulate the expression of Nrf2 to inhibit ROS accumulation in recipient cells.***A*, Western blot analysis of serum starved MEFs treated without or with EVs from ESCs for the indicated lengths of time, probed for Nrf2 and GSK3β when it is phosphorylated at Ser nine (p-GSK3β (Ser9)). Actin was the loading control. The data are representative of three independent experiments (n = 3). *B*, relative Nrf2 expression levels in (*A*) were determined using ImageJ software. (*C*) Images of MEFs treated without or with EVs from ESCs and stained with MitoSOX Red and Hoechst. Scale bar = 20 μm. The data are representatives of three independent experiments. (n = 100–130 cells; taken from 10 images per group). *D*, the fluorescence intensity of each cell in (*C*) was quantified using ImageJ software. *E*, MEFs were treated without or with EVs from ESCs and 1 μM MK-2206 (AKTi) and stained with MitoSOX Red and Hoechst. The fluorescence intensity of each cell was quantified using ImageJ software. The experiment was performed three independent times. (n = 60–100 cells; taken from 10 images per group). *F*, MEFs were treated without or with 3 μM GSK3β inhibitor CHIR99021 (GSK3βi) and stained with MitoSOX Red and Hoechst. The fluorescence intensity of each cell was quantified using ImageJ software. The experiment was performed three independent times. (n = 70–80 cells; taken from 10 images per group). The data shown in (*B*), (*D*), (*E*), and (*F*) were determined using Student’s *t* test; ∗∗∗∗*p* < 0.0001; ∗∗∗*p* < 0.001; and ns (not significant). Error bars indicate means ± standard deviations (SD).

GSK3β regulates the function of several proteins involved in cell cycle progression, including the transcription factors c-Myc and Nrf2 (nuclear factor erythroid 2-related factor) (66, 72, 73, 74). It is known to phosphorylate these transcription factors, which targets them for degradation in the proteasome. Therefore, we examined whether the EVs produced by ESCs increased the stability and accumulation of either c-Myc or Nrf2 in cells by inhibiting GSK3β activity. MEFs treated with EVs isolated from ESCs for as little as 1 h showed a significant increase in Nrf2 expression, but not c-Myc (Figs. 7, *A*, *B*, and S7*A*). The ability of the ESC-derived EVs to increase Nrf2 expression was then prevented by treating the cells with the AKT inhibitor MK-2206 (Fig. S7*B*).

**Figure 8:**
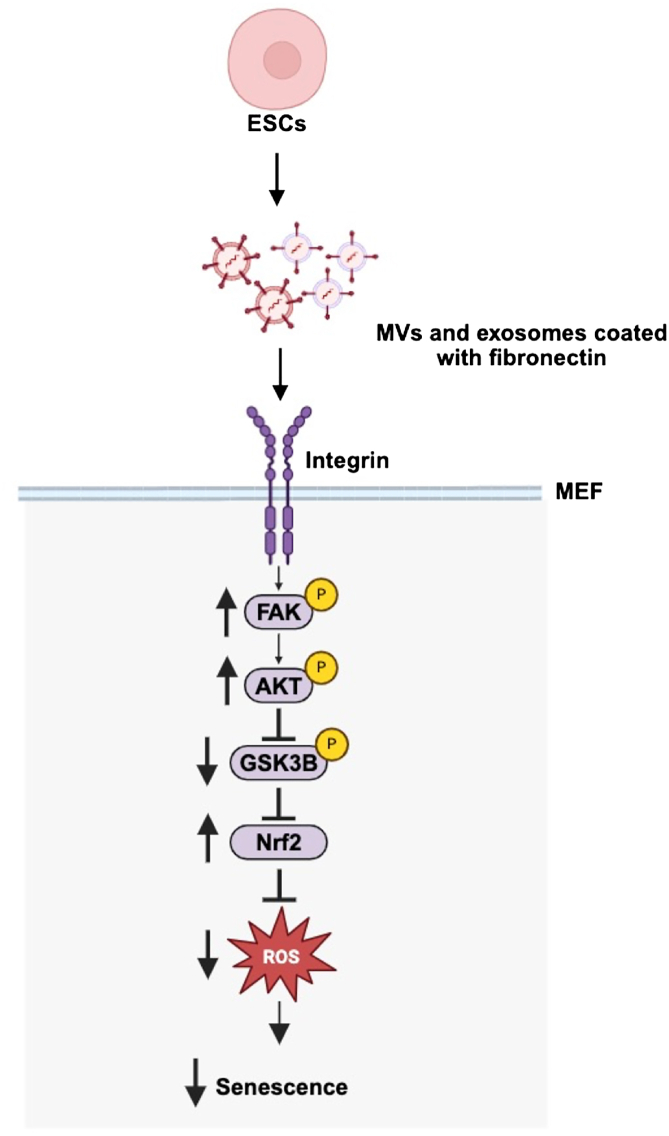
**Diagram showing how EVs produced by pluripotent ESCs delay cellular senescence.** The MVs and exosomes produced by ESCs are coated with fibronectin, which can engage and activate integrins expressed in target cells. This results in the activation of a signaling pathway that inhibits GSK3β, increases the stability of Nrf2, and suppresses the accumulation of ROS that would otherwise trigger cells to undergo senescence.

Nrf2 is a transcription factor involved in the regulation of several antioxidant genes, including superoxide dismutase (SOD) (75, 76). Oxidative stress caused by the accumulation of reactive oxygen species (ROS) has been reported to promote the induction of senescence (10, 77). We therefore determined whether ESC-derived EVs can reduce ROS levels in recipient cells using mitoSOX Red, a fluorescent dye that measures superoxide production in mitochondria (78). While untreated MEFs showed considerable amounts of ROS, when the cells were treated with EVs, there was a marked reduction in the amount of ROS produced by the cells (Fig. 7, *C* and *D*). Next, we examined if the suppression of ROS levels by EVs is dependent on their ability to promote the activation of AKT pathway. Treatment of serially passaged MEFs with different combinations of EVs and the AKT inhibitor blocked the ability of the EVs to suppress the accumulation of ROS (Figs. 7*E* and S7*C*). We also examined whether GSK3β inhibition alone could recapitulate the ability of ESC-derived EVs to suppress ROS levels. MEFs treated with the GSK3β inhibitor, CHIR99021, showed significantly reduced levels of ROS compared to the untreated cells (Figs. 7*F* and S7*D*). Taken together, these findings demonstrate that the EVs produced by ESCs alleviate ROS levels in MEFs through a fibronectin-dependent signaling pathway that leads to the activation of AKT and the upregulation of Nrf2, which suppresses GSK3β activity and delays cells from undergoing senescence (Fig. 8).

## Discussion

Cellular senescence is caused by the cumulative effects of various stresses that cells encounter throughout their lifetime (1, 2, 7, 9, 12). The unique secretome produced by senescent cells (*i.e.* SASP) promotes chronic inflammation and fibrosis, which further exacerbates the aging process by causing ‘young’ cells to undergo senescence (9, 11). The accumulation of senescent cells in organisms has been shown to contribute to the detrimental aspects of the aging process, as well as to the development of diseases including Alzheimer’s disease and cancer (12, 13, 79). Thus, establishing the underlying mechanisms that drive cellular senescence is critical, as this could lead to novel strategies to sustain the growth and proper functions of cells and block the detrimental effects associated with the secretome produced by aging and senescent cells.

There have been reports that EVs derived from stem cells or young cells have the potential to delay cellular senescence. For example, stem cell-derived EVs, or EVs in the plasma from young mice, were recently shown to rejuvenate aged recipient cells in culture, as well as in mouse models of aging (38, 39, 40, 80), although the mechanisms and signaling pathways essential for these beneficial effects have yet to be fully elucidated. In this report we demonstrate that EVs shed by pluripotent ESCs potently block primary fibroblasts and astrocytes from undergoing passage-induced senescence by triggering a signaling pathway that counteracts the detrimental effects of oxidative stress. It begins with fibronectin, bound to the outer surfaces of both MVs and exosomes, that engages integrins on cells. This then signals the activation of FAK and AKT, with activated AKT phosphorylating and inhibiting the kinase activity of GSK3β, increasing the stability of the transcription factor Nrf2 and leading to its accumulation in cells. Nrf2 promotes the expression of several antioxidant genes, thereby suppressing the accumulation of ROS and delaying cells from undergoing senescence (Fig. 8). Importantly, we further show that disrupting several steps in this pathway is sufficient to block the EV-promoted delay in senescence.

Because fibronectin associated with EVs derived from ESCs plays a critical role in their ability to delay senescence, it raised the question of whether the addition of free fibronectin to primary cells, or treating them with EVs from differentiated cells coated with fibronectin, would also delay their ability to undergo senescence. However, we found that neither of these conditions can fully recapitulate the functions of the stem cell vesicles. Previously, we determined that the relative amount of fibronectin associated with MVs and exosomes produced by ESCs was equivalent to ∼300 ng of fibronectin (37). When we treated MEFs with 300 ng of fibronectin through seven passages, this treatment was unable to prevent cells from undergoing senescence or maintain the expression levels of SIRT1 and NAMPT (Fig. S8, *A* and *B*). However, the addition of a large excess (∼16-fold; 5 μg) of fibronectin did result in a modest decrease in the number of senescent cells (Fig. S8*A*) and helped to maintain the expression of SIRT1 and NAMPT (Fig. S8*B*). We then collected the EVs produced by ESCs that have lost their pluripotency and undergone differentiation by removing them from their specialized culturing conditions that maintain stem cell pluripotency (37). The MVs and exosomes isolated from these differentiated cells lost their expression of fibronectin (Fig. S8*C*). However, similar to findings reported by others (81), combining these EVs with 300 ng of fibronectin results in its strong association with the vesicles (Fig. S8*D*), although they still failed to delay cellular senescence (Fig. S8, *E* and *G*). Collectively, these findings suggest that while the fibronectin associated with stem cell-derived EVs is important for activating signaling pathways in target cells that delay senescence, other cargo associated with the EVs (*i.e.* proteins, miRNAs, and lipids) are likely involved in promoting this effect.

In contrast to our elucidation of a fibronectin-promoted signaling pathway that leads to AKT activation and delays senescence, there have been suggestions that AKT activity can *promote* senescence (82, 83). These suggestions stem from findings that show the ectopic expression of oncogenic forms of the Ras GTPase in primary cells, which potently activate AKT, induces their senescence, *i.e.*, referred to as oncogene-induced senescence (84). Others have found that cells lacking PTEN, a negative regulator of PI3 kinase, as well as the ectopic expression of AKT in cells, can induce senescence *via* the activation of mTORC1, p53, and p21 (85). A possible explanation for these findings *versus* those that we present here may be the extent of AKT activation occurring in the different studies. In those cases where AKT was suggested to promote cellular senescence, its activity is quite high and sustained (82, 83, 85); whereas, the levels of AKT activity promoted by ESC-derived EVs are relatively modest and thus may preferentially activate the distinct signaling pathway that we show interferes with the establishment of a senescent phenotype. However, at the present time we cannot rule out the possibility that additional signals triggered by ESC-derived EVs work together with their activation of AKT to maintain the expression of key enzymes (*i.e.*, SIRT1 and NAMPT) and delay cellular senescence.

A critical outcome of the activation of AKT by ESC-derived EVs, which is essential for their ability to delay senescence, is the inactivation of GSK3β, a protein kinase best known for regulating the stability of several proteins involved in cell growth and survival (66, 70, 71). Upon phosphorylation by AKT, GSK3β is inactivated (70) which then leads to the increased expression of its substrates, including Nrf2 (72, 74). This transcription factor controls the expression of enzymes involved in limiting ROS levels in cells, including superoxide dismutase (SOD), catalase (CAT), and glutathione peroxidase (GPx) (75, 76). It is the accumulation of Nrf2 that occurs downstream of the signaling pathway triggered by ESC-derived EVs, which counteracts the buildup of ROS associated with replicative exhaustion and thereby allows cells to continue to grow while their untreated cellular counterparts undergo senescence. Thus, inhibiting GSK3β activity with the small molecule inhibitor CHIR99021 is sufficient to delay senescence. To our knowledge, this is the first demonstration that EVs from pluripotent cells inactivate GSK3β to delay senescence by preventing the accumulation of ROS.

Cellular senescence drives organismal aging by creating highly inflammatory micro-environments that promote aging-related processes and predispose organisms to various diseases and disorders (8, 13, 14, 79). Moreover, several types of primary cells cultured in the laboratory or isolated from patients for potential therapeutic use are difficult to grow and have finite lifespans, often making them extremely difficult to study (86, 87, 88, 89). Thus, understanding the molecular mechanisms that can delay cellular senescence is important for overcoming these challenges as well as for the development of new strategies for regenerative medicine and for the promotion of healthy aging. Here, we have described how EVs derived from ESCs are able to extend cellular lifespans through their initiation of an intricate signaling pathway that reduces ROS levels in cells which would otherwise succumb to senescence. In future work, we plan to determine how long the EVs from pluripotent ESCs can delay senescence in different cell types, and whether administering ESC-derived EVs to mice can delay aging-related phenotypes and diseases, including heart disease and neurodegeneration. However, because EVs contain a wide range of cargo, special attention will need to be given to any unexpected consequences that the EVs could potentially have in animal models, such as affecting tissue function or promoting cancer. Collectively, these efforts will broaden our understanding of the regenerative capabilities of EVs produced by stem cells, toward the goal of providing a new class of therapeutic agents against aging-related disorders and disease.

## Experimental procedures

### Cell lines

E14tg2a.4 mouse embryonic stem cells (ESCs) were cultured in 2i + LIF medium: a 1:1 mixture of Neurobasal and DMEM/F12 medium supplemented with 1 μM PD03259010, 3 μM CHIR99021, 1000 units/ml leukemia inhibitory factor, 1.5 × 10^-4^ M monothioglycerol, 2 mM glutamine, 0.05% bovine serum albumin, N-2 Supplement (100X, Thermo Fisher Scientific), and B27 Supplement (50X, Thermo Fisher Scientific). The cells were grown on tissue culture-treated plates coated with 0.1% gelatin. Mouse embryonic fibroblasts (MEFs) were derived from B6D2F1 mouse embryos and cultured in DMEM medium containing 10% fetal bovine serum (FBS). Mouse astrocytes (ScienCell) were isolated from the brains of C57BL/6 mice and cultured in Astrocyte Medium-animal (ScienCell) on tissue culture-treated plates coated with 0.01% poly-L-Lysine solution.

### Nanoparticle tracking analysis (NTA)

The conditioned medium collected from ESCs (∼5 × 10^5^ cells/cm^2^) was centrifuged twice at 1000*g* for 5 min at 4 °C to remove cells and debris. The partially clarified medium was injected into the NanoSight NS300 (Malvern Panalytical) beam path to capture movies of EVs under Brownian motion. Five 30-s movies of each sample were captured and used to determine the number and size of EVs present, and the results were averaged together.

### EV isolation

The conditioned medium collected from pluripotent ESCs (∼5 × 10^5^ cells/cm^2^) was centrifuged twice at 1000*g* for 5 min at 4 °C to remove cells and debris. The partially clarified medium was filtered using a 0.22 μm Steriflip filter unit (Millipore), followed by extensive washes with phosphate-buffered saline (PBS). The EVs larger than 0.22 μm retained by the filter are considered the microvesicle (MV) fraction. The filtrate (*i.e.* flowthrough) was subjected to ultracentrifugation at 100,000*g* for 5 h at 4 °C using a Type 45 Ti Rotor (Beckman) to pellet exosomes (EVs smaller than 0.22 μm). The isolated MV and exosome fractions were either resuspended in PBS (for biological assays) or lysed in lysis buffer (25 mM Tris, 100 mM NaCl, 1% Triton X-100, 1 mM EDTA, 1 μg/ml aprotinin, 1 μg/ml leupeptin, 1 mM β-glycerol phosphate, 1 mM DTT). The isolated EV fractions were stored at −80 °C for a maximum of 1 week before being used in experiments. For all cell-based experiments performed involving EVs, a total of 1 × 10^8^ vesicles were used per treatment (based on NTA), which is equivalent to 10 μg of EV protein lysate.

### EV transfer assay

The conditioned medium collected from ESCs (∼5 × 10^3^ cells/cm^2^) was centrifuged twice at 1000*g* for 5 min at 4 °C to remove cells and debris. FM1-43FX dye (Thermo Fisher Scientific) was added to the partially clarified medium to a final concentration of 5 μg/ml for 10 min. The medium was then subjected to the EV isolation procedure, as described above. The isolated MVs and exosomes labeled with the dye were resuspended in PBS and used to treat MEFs grown in 6-well plates for 1 h, at which point the cells were washed extensively with PBS, fixed with 4% formaldehyde, washed again extensively with PBS, stained with Hoechst, and placed in 90% glycerol. The cells were visualized using fluorescent microscopy and a 20x objective. All images were processed using ImageJ software.

### Sphere formation assay

ESCs and MEFs were plated in 6-well ultra-low attachment plates at a density of 5 × 10^3^ cells/cm^2^ and cultured in 2i + LIF medium. After 48 h, the cells were visualized using brightfield microscopy and a 20x objective. The number of spheres that formed for each cell type was determined.

### Alkaline phosphatase (AP) activity assay

ESCs and MEFs were plated in 6-well plates coated with 0.1% gelatin at a density of 5 × 10^3^ cells/cm^2^ and cultured in 2i + LIF medium. After 48 h, the cells were fixed with 4% formaldehyde and stained with VECTOR Red Alkaline Phosphatase Substrate solution (Vector Laboratories) according to the instructions provided by the manufacturer. The cells were rinsed with PBS, placed in 90% glycerol, and visualized using brightfield microscopy and a 20x objective. The percentage of alkaline phosphatase-positive cells (*i.e.* cells that stained red) was determined by dividing the number of stained cells by the total number of cells.

### Cell growth assay

Passage 3 MEFs and astrocytes were plated in 60 mm plates at a density of 5 × 10^3^ cells/cm^2^ and cultured in their growth medium supplemented with different combinations of PBS, DMSO, MVs and exosomes (1.0 × 10^8^ MVs and exosomes, or the equivalent of 10 μg of EV protein lysate), MK-2206 (1 μM), FAK inhibitor III (5 μM), and CHIR99021 (3 μM), as indicated. Every third day, the cells were trypsinized, counted, and re-plated at the same density (5 × 10^3^ cells/cm^2^) in new 60 mm plates with fresh medium and treatments. The cells were routinely passaged this way (*i.e.* serially passaged) until the control group of cells stopped growing. The cells at each passage were also collected and used for Western blot analysis and SA-β-galactosidase activity assays as described below.

### Senescence-associated (SA) β-galactosidase activity assay

Cells that had been treated and/or passaged as indicated were plated in 60 mm plates at a density of 5 × 10^3^ cells/cm^2^. The next day, the cells were stained with the Senescence β-Galactosidase Staining Kit (Cell Signaling Technology) according to the instructions provided by the manufacturer. Briefly, the cells were washed twice with PBS, fixed with the SA-β-Galactosidase fixing solution, washed again with PBS, and then incubated with staining solution for 24 h at 37 °C. The plates were sealed with parafilm during this step to prevent evaporation and oxidation of the staining solution. The cells were then washed with PBS and visualized using brightfield microscopy and a 20x objective. Approximately 200 cells were counted for each condition and the percentage of β-gal-positive cells (*i.e.* cells that stained blue) was determined by dividing the total number of stained cells by the total number of cells.

### Western blot/Immunoblot analysis

Protein concentrations of cell, MV, and exosome lysates were determined using the Bio-Rad Protein Assay Dye (Bio-Rad) and a spectrophotometer (Beckman). The lysates were normalized based on protein concentration and resolved on 4 to 20% SDS-PAGE gels (Invitrogen). The gels were transferred to PVDF membranes (Thermo Fisher Scientific), and the membranes were blocked with 10% bovine serum albumin (BSA) in 20 mM Tris, 135 mM NaCl, and 0.02% Tween 20 (TBST) for 1 h at room temperature. Primary antibodies diluted in TBST were added to the blots and incubated overnight at 4 °C. The membranes were extensively washed with TBST and incubated with HRP-conjugated secondary antibodies diluted in TBST for 1 h at room temperature. The membranes were again washed with TBST, before being exposed to ECL reagents (Bio-Rad). Images of the membranes were obtained using X-ray films (The Lab Depot). Molecular weight markers are included along the right side of all panels involving Western blots.

### Trypsin digestion assays

Batches of intact MVs and exosomes isolated from ESCs were evenly divided into two. One sample was left untreated while the other sample was treated with trypsin (400 μg/ml) for 15 min at which point soybean trypsin inhibitor (1 mg/ml) was added to the EVs. The samples were either resuspended in PBS (for biological assays) or lysis buffer (for Western blot analysis).

### Inhibitor and RGD peptide treatments

As indicated, cells were treated with 1 μM MK-2206 (the AKT inhibitor; AKTi) reconstituted in DMSO, 5 μM FAK inhibitor III (the FAK inhibitor; FAKi) reconstituted in DMSO, 3 μM CHIR99021 (the GSK3β inhibitor; GSK3βi) reconstituted in DMSO, and 100 μg/ml RGD peptide reconstituted in PBS. An equivalent volume of appropriate vehicle (either DMSO or PBS) was added to the untreated control groups in all experiments involving these inhibitors.

### Signaling experiments in MEFs

MEFs plated in 6-well dishes at a density of 5 × 10^3^ cells/cm^2^ were washed 3 times with PBS and placed in serum-free DMEM containing various combinations of inhibitors and vehicle controls. Approximately 16 h later, the cells were treated without or with MVs and exosomes isolated from ESCs for the indicated lengths of time, and then lysed with lysis buffer. In certain cases, MVs and exosomes from ESCs were first treated with trypsin before being used to treat MEFs. The lysates were subjected to Western blot analysis.

### MitoSOX red staining assay

MEFs were plated in 35 mm plates at a density of 5 × 10^3^ cells/cm^2^ and cultured in the growth medium supplemented with the indicated inhibitors and EVs (∼10^8^ vesicles) isolated from ESCs for 24 h. The cells were then incubated for 10 min with 100 nM of MitoSOX Red reagent (Thermo Fisher Scientific). The cells were then visualized using fluorescent microscopy and a 20x objective. All images were processed using ImageJ software.

### Negative stain electron microscopy

The conditioned medium collected from cultures of ESCs was centrifuged twice at 1000*g* for 5 min at 4 °C to remove cells and debris. 10 μl of the partially clarified medium was placed on a copper grid and the excess medium was removed by blotting with filter paper. 10 μl of 2.0% uranyl acetate was added to the grid for 5 min before the excess liquid was blotted with the filter paper. The grid was then visualized using electron microscopy.

### Attaching purified fibronectin to EVs from differentiated cells

E14tg2a.4 mouse embryonic stem cells (ESCs) were cultured in growth medium lacking PD03259010 and CHIR99021 for 5 days, which results in their losing pluripotency and undergoing cellular differentiation (37). The MVs and exosomes isolated from these differentiated cells were incubated with 300 ng of purified fibronectin at room temperature for 15 min. The EV and fibronectin mixture was then ultracentrifuged at 100,000*g* for 2 h at 4 °C using the TLA100.4 rotor (Beckman). The pelleted EVs were resuspended in either lysis buffer for Western blot analysis or PBS for the treatment of MEFs.

### Quantification and statistical analysis

All experiments were performed at least three independent times, and the statistical significance of the results was determined using either Student’s *t* test or analysis of variance (ANOVA), as needed; ∗∗∗∗*p* < 0.0001; ∗∗∗*p* < 0.001; ∗∗*p* < 0.01; ∗*p* < 0.05; and ns (not significant). Error bars indicate means ± standard deviations (SD).

## Data availability

All data are contained within the manuscript.

## Supporting information

This article contains supporting information.

## Conflict of interest

The authors declare that they have no conflicts of interest with the contents of this article.

## Supporting information

Supporting Figures

Key Resource Table
