# Supplementary material for: Embryonic stem cell-derived extracellular vesicles delay cellular senescence by inhibiting oxidative stress[image]
Source: J Biol Chem. 2025 Oct 14;301(12):110821. doi: 10.1016/j.jbc.2025.110821 (PMC12648614; doi:10.1016/j.jbc.2025.110821)
Supplement: Supporting Figures [file mmc1.pdf]

Embryonic stem cell-derived extracellular vesicles delay cellular senescence by inhibiting oxidative stress

Shun Enomoto

Yun Ha Hur

Tatiana Solodova

Jacob Neumann

Richard A. Cerione

Marc A. Antonyak

Figures S1 – S8

Supplementary Figure Legends

Figure S1. Characterization of ESCs and MEFs, related to Figure 1.

(A) Images of ESCs and MEFs. Scale bar = 50 μm. The data are representative of 3 independent experiments (n = 3). **(B)** Western blot of ESCs and MEFs probed for the stem cell markers Oct3/4, Nanog, and Sox2, and the fibroblast marker Thy1. Hsp90 was the loading control. The data are representative of 3 independent experiments (n = 3). **(C)** Images of sphere formation assays performed on ESCs and MEFs. Scale bar = 50 μm. The data are representative of 3 independent experiments (n = 3). **(D)** The number of spheres that formed in (C) were determined. **(E)** Images of Alkaline Phosphatase (AP) activity assays performed on ESCs and MEFs. Cells that stained red were positive for AP activity. Scale bar = 50 μm. The data are representative of 6 independent experiments. (n = 6). **(F)** The percentage of AP-positive cells in (E) were determined. **(G)** Images of β-galactosidase activity assays performed on MEFs that had been serially passaged 8 times (passage 8; P8), and ESCs that had been passaged 8 or 25 times (passage 8 or 25; P8 or P25, respectively). Scale bar = 50 μm. The data are representative of 3 independent experiments (n = 3). **(H)** The percentage of β-galactosidase positive cells in (G) was determined. The data shown in (D), (F), and (H) were determined using Student's t test; ****$p < 0.0001$; and ***$p < 0.001$. Error bars indicate means ± standard deviations (SD).

Figure S2. EVs derived from ESCs inhibit passage-induced cellular senescence, related to Figure 2.

(A) Images of Passage 7 (P7) MEFs that had been treated without or with EVs isolated from ESCs. Scale bar = 25 μm. The data are representative of 3 independent experiments (n = 3). **(B)** Images β-galactosidase activity assays performed on MEFs that had been treated without or with EVs isolated from ESCs for 2 or 8 passages (P2 and P8). Scale bar = 50 μm. The data are representative of 4 independent experiments (n = 4). **(C)** Western blot of MEFs treated without or with EVs from ESCs for 4 and 7 passages (P4 and P7), probed for the senescence markers SIRT1 and NAMPT. Actin was the loading control. The data are representative of 3 independent experiments (n = 3). **(D)** Images of β-galactosidase activity assays performed on astrocytes treated without or with EVs isolated from ESCs for 8 passages (P8). Scale bar = 50 μm. The data are representative of 3 independent

experiments (n = 3). **(E)** Western blot of MEFs treated without or with EVs from ESCs for 4 and 7 passages (P4 and P7), probed for the fibroblast marker Thy1. Vinculin was the loading control. The data are representative of 3 independent experiments (n = 3). **(F)** Western blot of astrocytes treated without or with EVs from ESCs for 4 and 7 passages (P4 and P7), probed for the astrocyte marker GFAP. Actin was the loading control. The data are representative of 3 independent experiments (n = 3).

Figure S3. EVs derived from ESCs activate AKT to delay senescence in recipient fibroblasts, related to Figure 3.

(A and B) Western blot of serum starved MEFs that had been treated without or with MVs or exosomes (EXO) isolated from ESCs for the indicated lengths of time, probed for AKT when it is phosphorylated at (A) Thr 308 (p-AKT (T308)) and (B) Ser 473 (p-AKT (S473)). Vinculin and Actin were the loading controls. The data are representative of 3 independent experiments (n = 3). **(C)** Images of β-galactosidase activity assays performed on MEFs treated without or with EVs from ESCs and MK-2206 (AKTi) for 8 passages (P8). Scale bar = 50 μm. The data are representative of 3 independent experiments (n = 3).

Figure S4. Fibronectin coating the surface of EVs from ESCs activate FAK in recipient cells, related to Figure 4.

(A) Western blot of intact MVs and exosomes isolated from ESCs that were treated without or with trypsin. The cells (WCL) and vesicles were probed for fibronectin, the MV marker Hsp90, ubiquitin, and the exosome marker CD81. The data are representative of 3 independent experiments (n = 3). **(B)** Western blot of serum starved MEFs that had been treated without or with EVs isolated from ESCs that were treated without or with trypsin for the indicated lengths of time. The cells were probed for AKT when it is phosphorylated at either Thr 308 (p-AKT (T308)) or Ser 473 (p-AKT (S473)). Actin was the loading control. The data are representative of 3 independent experiments (n = 3).

Figure S5. EV-induced FAK activation is necessary to delay senescence, related to Figure 5.

(A) Images of β-galactosidase activity assays performed on MEFs treated without or with EVs from ESCs and 5μM FAK inhibitor III (FAKi) for 8 passages (P8). Scale bar = 50 μm. The data are representative of 3 independent experiments (n = 3).

Figure S6. EVs produced by ESCs inhibit GSK3β activity via activating integrin, FAK, and AKT, related to Figure 6.

(A) Western blot of serum starved MEFs treated without or with EVs from ESCs and 100μg RGD peptide, probed for AKT when it is phosphorylated at Ser 473 (p-AKT (Ser473)) and GSK3β when it is phosphorylated at Ser 9 (p-GSK3β (Ser9)). Actin was the loading control. The data are representative of 3 independent experiments (n = 3). **(B)** Western blot of serum starved MEFs treated without or with EVs from ESCs and 1μM FAK inhibitor III, probed for AKT when it is phosphorylated at Ser 473 (p-AKT (Ser473)) and GSK3β when it is phosphorylated at Ser 9 (p-GSK3β (Ser9)). Actin was the loading control. The data are representative of 3 independent experiments (n = 3). **(C)** Images of β-galactosidase activity assays performed on MEFs treated without or with 3μM GSK3β inhibitor CHIR99021 (GSK3βi) for 8 passages (P8). Scale bar = 20 μm. The data are representative of 3 independent experiments (n = 3).

Figure S7. EVs-mediated upregulation of Nrf2 is dependent on AKT activity, related to Figure 7.

(A) Western blot of serum starved MEFs treated without or with EVs from ESCs for the indicated lengths of time, probed for c-myc. Actin was the loading control. The data are representative of 3 independent experiments (n = 3). **(B)** Western blot of serum starved MEFs treated with EVs from ESCs and 1μM MK-2206, probed for Nrf2 and GSK3β when it is phosphorylated at Ser 9 (p-GSK3β (Ser9)). Actin was the loading control. The data are representative of 3 independent experiments (n = 3). **(C)** Images of MEFs treated without or with EVs from ESCs and 1μM MK-2206 (AKTi) and stained with MitoSOX Red and Hoechst. Scale bar = 20 μm. The data are representatives of 3 independent experiments. (n = 60-100 cells; taken from 10 images per group). **(D)** Images of

MEFs treated without or with 3μM GSK3β inhibitor CHIR99021 (GSK3βi) and stained with MitoSOX Red and Hoechst. Scale bar = 20 μm. The data are representatives of 3 independent experiments. (n = 70-80 cells; taken from 10 images per group).

Figure S8. Treating MEFs with purified fibronectin cannot recapitulate the effect of EVs on delay cellular senescence.

(A) Passage 3 MEFs were treated without or with 300 ng or 5 μg of purified fibronectin. The cells were trypsinized, counted, and re-plated ($5x10^3$ cells/cm^2) in fresh medium supplemented without or with fibronectin every three days until the MEFs were passaged 7 times (P7). The cells were subjected to β-galactosidase activity assays and the percentage of senescent cells was determined. **(B)** Western blot of MEFs treated without or with 300 ng or 5 μg of purified fibronectin for 3, 5, and 7 passages (P3, P5, P7), probed for the senescence markers SIRT1 and NAMPT. Actin was the loading control. **(C)** Western blot of the MVs and exosomes (EXO) isolated from pluripotent ESCs and their differentiated counterparts (*Differentiated*). These samples, we well as the cells (WCL) that produced the EVs, were probed for the MV marker VDAC and fibronectin. **(D)** MVs and exosomes isolated from the differentiated cells of ESCs were incubated with 300 ng of purified fibronectin (FN), ultracentrifuged (100,000 xg) to pellet the vesicles, and Western blotted for the MV marker VDAC, exosome marker CD81, and fibronectin. **(E)** Passage 3 MEFs were treated with EVs isolated from the differentiated cells that had been incubated without or with 300 ng of purified fibronectin. The cells were trypsinized, counted, and re-plated ($5x10^3$ cells/cm^2) in fresh medium supplemented without or with EVs every three days until the MEFs were passaged 8 times (P8). β-galactosidase activity assays were performed on the cells, and the percentage of senescent cells was determined. **(F)** Western blot of MEFs treated EVs isolated from the differentiated cells that had been incubated without or with 300 ng of purified fibronectin. The cells were probed for the senescence markers SIRT1 and NAMPT. Actin was the loading control. The data are representative of 3 independent experiments (n = 3). The data shown in (E) was determined using Student's t test; ns (not significant). Error bars indicate means ± standard deviations (SD).

Figure S1

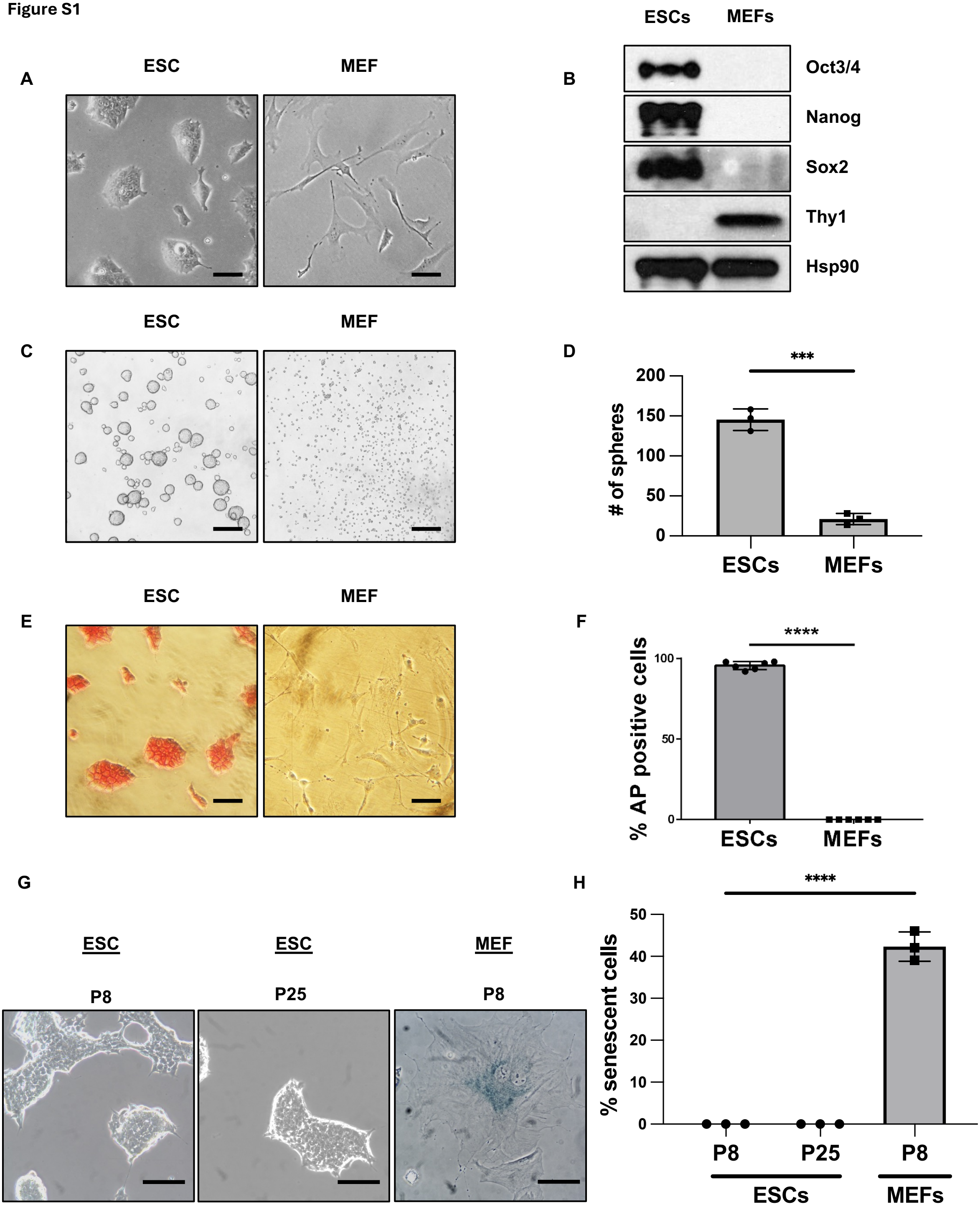
A
ESC
MEF
B
ESCs
MEFs
Oct3/4
Nanog
Sox2
Thy1
Hsp90
C
ESC
MEF
D
***
# of spheres
200
150
100
50
0
ESCs
MEFs
E
ESC
MEF
F
****
% AP positive cells
100
50
0
ESCs
MEFs
G
ESC
P8
ESC
P25
MEF
P8
H
****
% senescent cells
50
40
30
20
10
0
P8
P25
P8
ESCs
MEFs

Figure S2

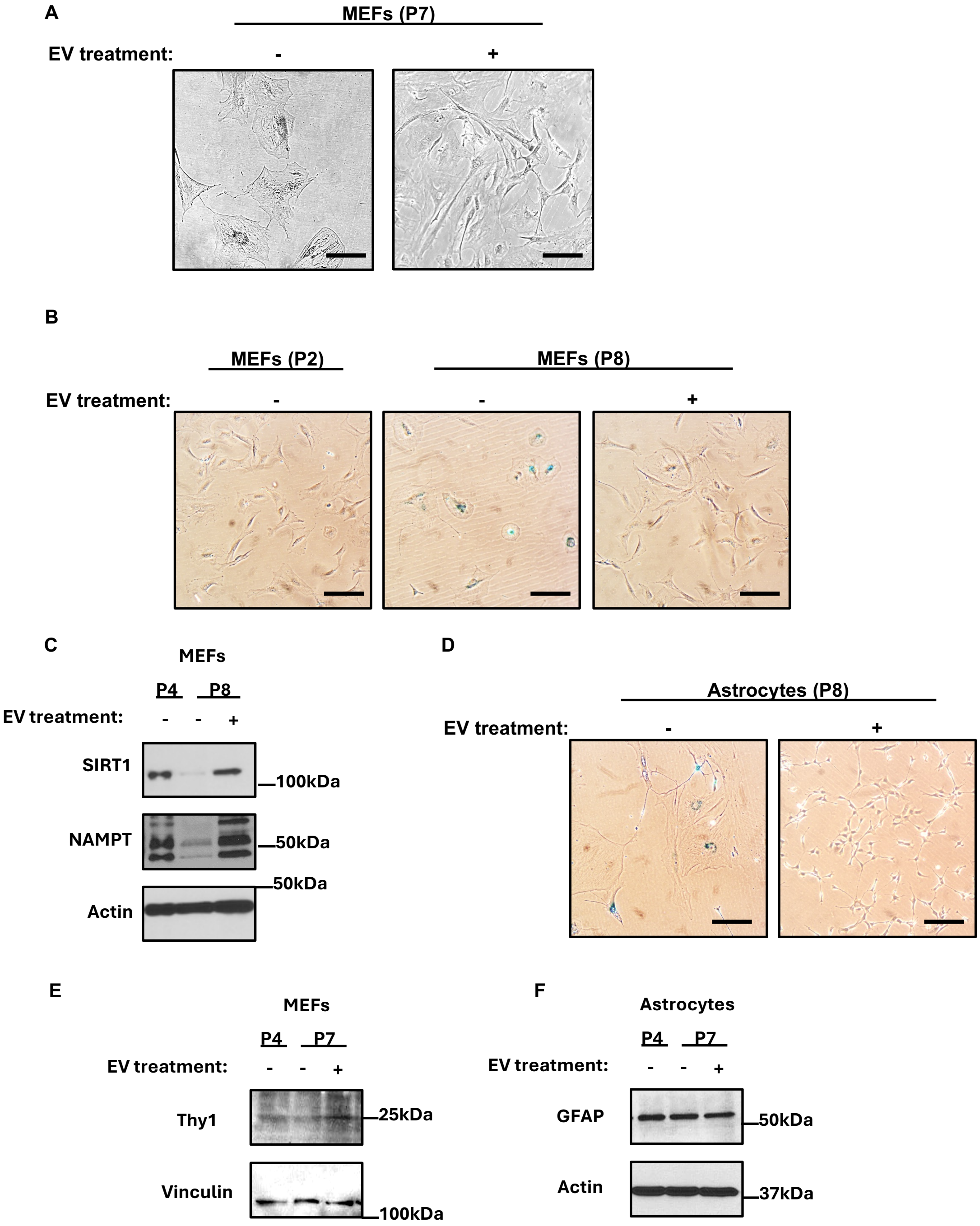

A
MEFs (P7)
EV treatment:
-
+
B
MEFs (P2)
MEFs (P8)
EV treatment:
-
-
+
C
MEFs
P4
P8
EV treatment:
-
-
+
SIRT1
100kDa
NAMPT
50kDa
50kDa
Actin
D
Astrocytes (P8)
EV treatment:
-
+
E
MEFs
P4
P7
EV treatment:
-
-
+
Thy1
25kDa
Vinculin
100kDa
F
Astrocytes
P4
P7
EV treatment:
-
-
+
GFAP
50kDa
Actin
37kDa

Figure S3

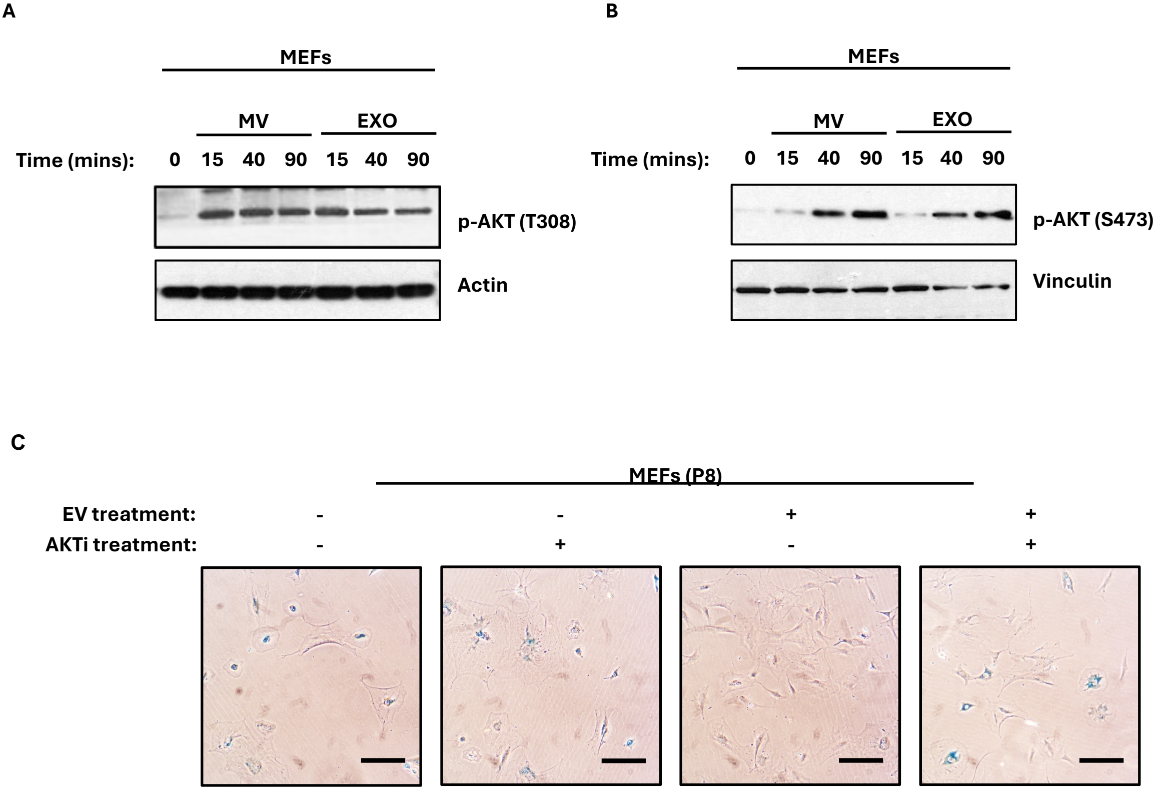
A
MEFs
MV
EXO
Time (mins):
0
15
40
90
15
40
90
p-AKT (T308)
Actin
B
MEFs
MV
EXO
Time (mins):
0
15
40
90
15
40
90
p-AKT (S473)
Vinculin
C
MEFs (P8)
EV treatment:
-
-
+
+
AKTi treatment:
-
+
-
+

Figure S4

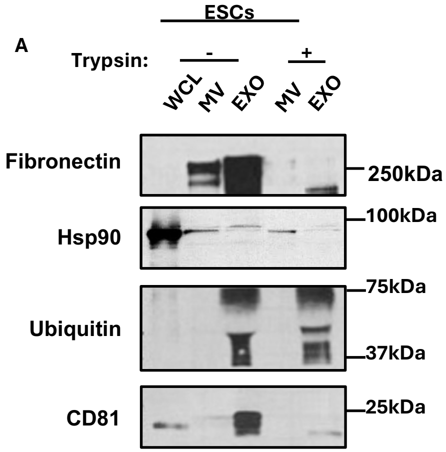
A
ESCs
Trypsin:
-
+
WCL
MV
EXO
MV
EXO
Fibronectin
250kDa
Hsp90
100kDa
Ubiquitin
75kDa
37kDa
CD81
25kDa

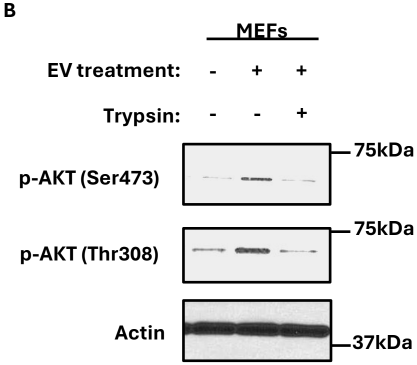
B
MEFs
EV treatment: - + +
Trypsin: - - +
p-AKT (Ser473)
75kDa
p-AKT (Thr308)
75kDa
Actin
37kDa

Figure S5

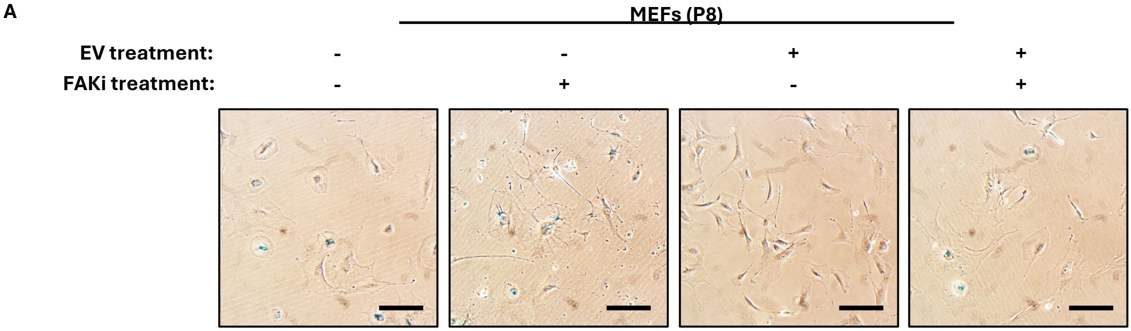

Figure S6

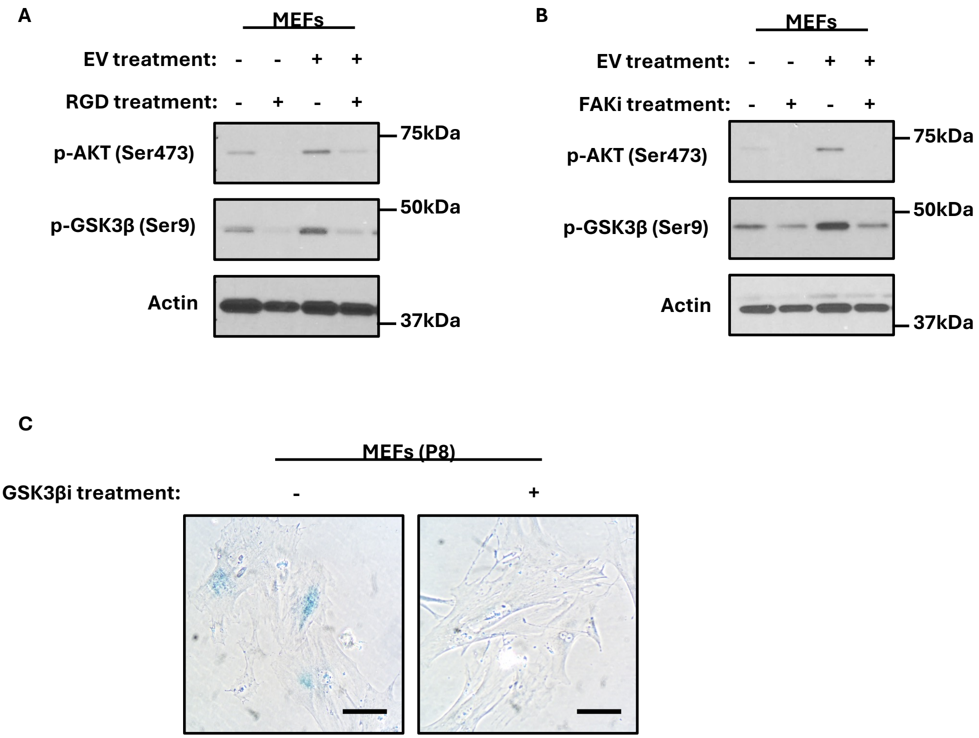
A
MEFs
EV treatment: - - + +
RGD treatment: - + - +
p-AKT (Ser473)
p-GSK3β (Ser9)
Actin
75kDa
50kDa
37kDa
B
MEFs
EV treatment: - - + +
FAKi treatment: - + - +
p-AKT (Ser473)
p-GSK3β (Ser9)
Actin
75kDa
50kDa
37kDa
C
MEFs (P8)
GSK3βi treatment: - +

Figure S7

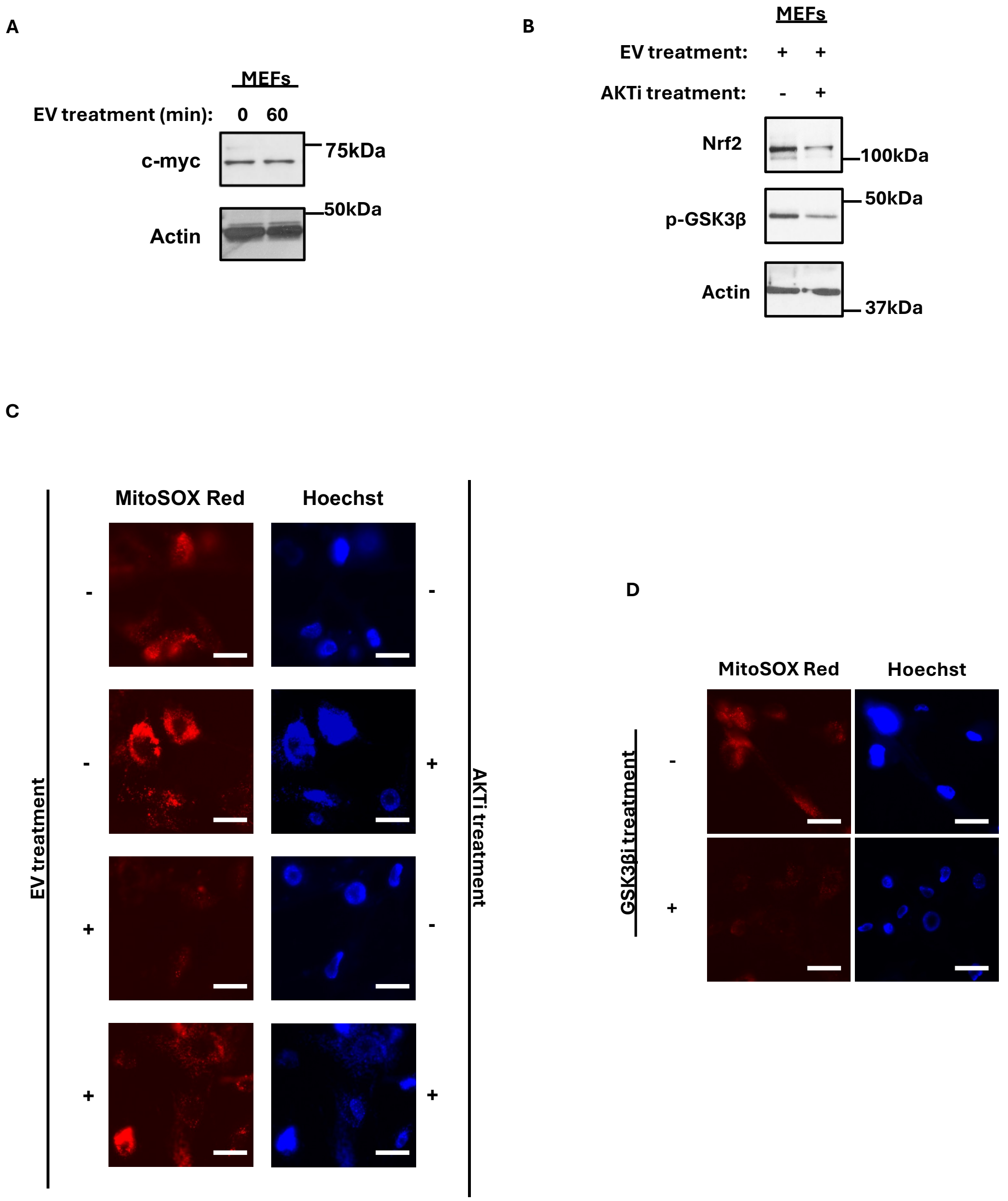
A
MEFs
EV treatment (min): 0 60
c-myc
75kDa
Actin
50kDa
B
MEFs
EV treatment: + +
AKTi treatment: - +
Nrf2
100kDa
p-GSK3β
50kDa
Actin
37kDa
C
MitoSOX Red
Hoechst
EV treatment
AKTi treatment
D
MitoSOX Red
Hoechst
GSK3βi treatment

Figure S8

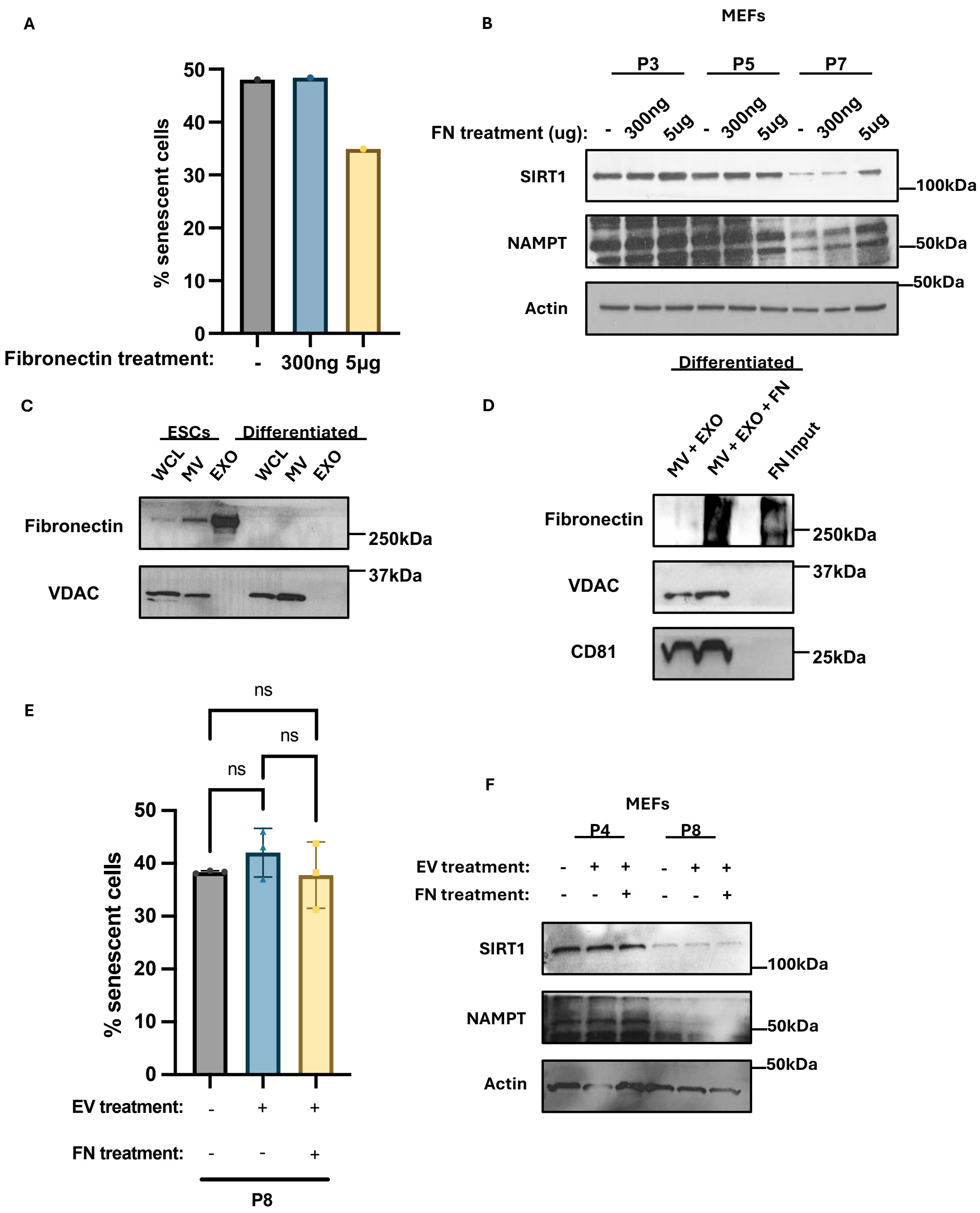
A
% senescent cells
50
40
30
20
10
0
Fibronectin treatment:
-
300ng
5μg
B
MEFs
P3
P5
P7
FN treatment (ug):
-
300ng
5ug
-
300ng
5ug
-
300ng
5ug
SIRT1
100kDa
NAMPT
50kDa
50kDa
Actin
C
ESCs
Differentiated
WCL
MV
EXO
WCL
MV
EXO
Fibronectin
250kDa
37kDa
VDAC
D
Differentiated
MV + EXO
MV + EXO + FN
FN Input
Fibronectin
250kDa
VDAC
37kDa
CD81
25kDa
E
ns
ns
ns
% senescent cells
50
40
30
20
10
0
EV treatment:
-
+
+
FN treatment:
-
-
+
P8
F
MEFs
P4
P8
EV treatment:
-
+
+
-
+
+
FN treatment:
-
-
+
-
-
+
SIRT1
100kDa
NAMPT
50kDa
50kDa
Actin
