# Supplementary material for: Embryonic stem cell-derived extracellular vesicles delay cellular senescence by inhibiting oxidative stress[image]
Source: J Biol Chem. 2025 Oct 14;301(12):110821. doi: 10.1016/j.jbc.2025.110821 (PMC12648614; doi:10.1016/j.jbc.2025.110821)
Supplement: Key Resource Table [file mmc2.docx]

**Key Resource Table**

| **REARGENT OR RESOURCE** | **SOURCE** | **IDENTIFIER** |
| --- | --- | --- |
| **Antibodies** |  |  |
| Hsp90 | Cell Signaling Technology | 4877 |
| IκBα | Cell Signaling Technology | 9242 |
| CD81 | Cell Signaling Technology | 10037 |
| VDAC | Cell Signaling Technology | 4661 |
| LAMP1 | Cell Signaling Technology | 15665 |
| SIRT1 | Cell Signaling Technology | 8469 |
| Actin | Cell Signaling Technology | 3700 |
| GFAP | Cell Signaling Technology | 12389 |
| Phospho-AKT (Thr308) | Cell Signaling Technology | 2965 |
| Phospho-AKT (Ser473) | Cell Signaling Technology | 9271 |
| AKT | Cell Signaling Technology | 4691 |
| NAMPT | ProteinTech | 11776-1-AP |
| Fibronectin | Sigma-Aldrich | F3648 |
| Phospho-FAK (Tyr397) | Cell Signaling Technology | 3283 |
| FAK | Cell Signaling Technology | 3285 |
| Phospho-GSK3β (Ser9) | Cell Signaling Technology | 9336 |
| GSK3β | Cell Signaling Technology | 9315 |
| Nrf2 | Cell Signaling Technology | 12721 |
| Oct3/4 | Santa Cruz Biotechnology | sc-5279 |
| Nanog | Abcam | ab80892 |
| Sox2 | Cell Signaling Technology | 4900 |
| Thy1 | Cell Signaling Technology | 13801 |
| Vinculin | Cell Signaling Technology | 13901 |
| Ubiquitin | Santa Cruz Biotechnology | sc-8017 |
| c-myc | Cell Signaling Technology | 5605 |
| Rabbit IgG-HRP conjugate antibody | Cell Signaling Technology | 7074 |
| Mouse IgG-HRP conjugate antibody | Cell Signaling Technology | 7076 |
| **Chemicals, Peptides, and Recombinant Proteins** | |  |
| Aprotinin | Sigma-Aldrich | 10236624001 |
| Astrocyte Medium-animal | ScienCell | 1831 |
| β-glycerophosphate | Calbiochem | 2768360 |
| B-27 Supplement | Thermo Fisher Scientific | 17504044 |
| Bio-Rad protein assay | Bio-Rad | 5000006 |
| CHIR99021 | MedChemExpress | HY-10182 |
| Dimethyl sulfoxide (DMSO) | Sigma-Aldrich | D8418 |
| Dithiothreitol (DTT) | Sigma-Aldrich | 10197777001 |
| DMEM | Gibco | 11965-092 |
| DMEM/F12 | Gibco | 12634-010 |
| Western Lighting Plus-ECL | PerkinElmer | NEL105001EA |
| FAK inhibitor III | Sigma-Aldrich | 5040450001 |
| Fetal bovine serum (FBS) | Gibco | 10437028 |
| Fibronectin | Millipore | 341631 |
| FM1-43FX | Thermo Fisher Scientific | F35355 |
| GRGDSP | Sigma-Aldrich | SCP0157 |
| Hoechst 33342 | Thermo Fisher Scientific | 2189158 |
| L-Ascorbic acid | Millipore | 1831 |
| Leukemia inhibitory factor | Santa Cruz Biotechnology | sc-4989 |
| Leupeptin | Sigma-Aldrich | L9783 |
| L-Glutamine | Sigma-Aldrich | G8540 |
| MK-2206 | MedChemExpress | HY-10358 |
| Monothioglycerol | Sigma-Aldrich | M6145 |
| N-2 supplement | Thermo Fisher Scientific | 17502048 |
| N-acetyl-L-cysteine | Calbiochem | A9165 |
| Neurobasal medium | Gibco | 21103049 |
| Phosphate buffered saline tablets | VWR | VERVE404 |
| PD03259010 | MedChemExpress | HY-10254 |
| Poly-L-Lysine | Sigma-Aldrich | P4707 |
| Pen-Strep | Gibco | 15140122 |
| Triton X100 | Sigma-Aldrich | 9036-19-5 |
| Trypsin-EDTA (0.05%) | Gibco | 25300054 |
| **Critical Commercial Assays** |  |  |
| VECTOR Red Alkaline phosphatase substrate kit | Vector Laboratories | SK-5100 |
| MitoSOX Mitochondrial Superoxide Indicators | Thermo Fisher Scientific | M36008 |
| Senescence β-Galactosidase Staining Kit | Cell Signaling Technology | 9860 |
| **Cell Lines** |  |  |
| Mouse: E14tg2a.4 | ATCC | CRL-1821 |
| Mouse: Mouse Embryonic Fibroblasts | Cornell Stem Cell and Transgenic Core Facility | N/A |
| Mouse: Astrocytes | ScienCell | M1800 |
| **Software and Algorithms** |  |  |
| ImageJ | NIH | N/A |
| Prism | Graphpad | N/A |
| Biorender | Biorender | N/A |
| **Other Equipment** |  |  |
| 0.22 µm Steriflip filter | Millipore | SEM1M179M6 |
| Fluorescence microscope | KEYENCE | BZ-X800 |
| NanoDrop spectrophotometer | Thermo Fisher Scientific | 840-274200 |
| NanoSight NS300 | Malvern | N/A |
| Type 45 Ti rotor | Beckman Coulter | N/A |
| TLA100.4 rotor | Beckman Coulter | N/A |
| SW 41 Ti rotor | Beckman Coulter | N/A |
| Ultra-low attachment plate (6 well) | Corning | 3471 |
| Ultracentrifuge | Beckman Coulter | N/A |
